# Resource-Aware Safety-First Active Sensor Acquisition with Few-Shot Commissioning for Edge Fault Warning

**DOI:** 10.3390/s26165065

**Published:** 2026-08-10

**Authors:** Yanbo Bian, Hengrui Yu, Dengyuan Liu, Weiye Cai, Zhihai Wang

**Affiliations:** 1 Weihai International College, Beijing Jiaotong University, Weihai 264401, China; 2 School of Computer Science and Technology, Beijing Jiaotong University, Beijing 100044, China

**Keywords:** active feature acquisition, adaptive sensing, edge intelligence, few-shot commissioning, device shift, fault diagnosis

## Abstract

Edge monitoring balances diagnostic information against high-rate sensing costs. We formulate active acquisition: Low-cost signals screen each window, while high-information channels are acquired when alarms require confirmation or the screen is uncertain or out of distribution. The confirmation-complete controller includes an optional watchdog to bound blind intervals. On a five-stress benchmark, it retains 98.42% Macro-F1 versus 99.02% for always-full sensing, with 77.59% activation and 19.27% lower normalized resource costs. CWRU evaluation yields 99.25 ± 0.41% Macro-F1 at 83.30% activation. Paderborn condition-transfer evaluation matches always-full sensing at 91.08 ± 17.55% but activates every window; few-shot commissioning increases 15-bearing performance from 58.42 ± 18.60% to 76.52%. When each damage type is excluded from training and bearing identities are separated, the policy activates for 85.25 ± 18.84% of unseen-fault windows versus 83.61 ± 25.69% for a learned error gate. Across 40 healthy-to-unseen replays, 95% activate within five decision windows; an H=10 watchdog limits the inactive run to nine. These rates measure escalation, not unknown-class identification. An ESP32 pilot verifies telemetry feasibility but not sensor-rail power or end-to-end diagnosis. Reduced sensing is credible when the confirm stage transfers; otherwise, commissioning or conservative acquisition is required.

## 1. Introduction

Edge intelligence moves sensing, inference, and decision-making from cloud servers to resource-constrained devices [1]. In condition monitoring, this shift creates a coupled systems problem: A device must decide not only how to classify a signal but also which signal is worth acquiring. Continuously sampling every high-rate channel can dominate storage, communication, and sensing duty even when most monitoring windows are routine. Low-cost current or temperature measurements are attractive for continuous screening, whereas vibration and acoustic measurements provide richer fault information at a higher acquisition cost.

Learning-based fault diagnosis has improved classification through deep networks, transfer learning, edge inference, and multi-sensor fusion [2,3,4,5,6]. These methods generally begin after the required channels have been collected. Active feature acquisition instead treats measurement as an instance-wise sequential decision: each additional observation should justify its cost by improving the current prediction [7,8,9,10]. Dynamic neural inference similarly adapts computation to sample difficulty or resource budgets [11,12,13]. Sensor acquisition differs from layer skipping because an unmeasured physical channel cannot be reconstructed by merely allocating more computation.

This paper studies resource-aware, safety-first active sensor acquisition for edge fault warnings. Unlike fusion-centric diagnosis frameworks, the proposed controller acts before full sensing occurs. Low-cost features screen each window, and high-information features are activated whenever the screen raises an alarm, is uncertain, or lies far from source prototypes. The design is confirmation-complete: A cheap-stage fault prediction is a request for verification rather than a final warning. A periodic watchdog can additionally bound the interval for faults that have no cheap-channel signature. Because this conservatism only helps when the confirm-stage classifier transfers, this paper explicitly evaluates both operating-condition shifts and unseen-device identity shifts and then measures how much labelled commissioning data are needed to recover useful performance.

The main contributions are as follows:

- A resource-aware acquisition formulation that separates physical observation costs from the cost of classifying already observed data and reports conclusions over a range of high-to-low sensing-cost ratios.
- A confirmation-complete screen–confirm controller combining alarm status, risk, classifier margin, prototype distance, and an optional periodic watchdog, with explicit escalation and reaction-time bounds.
- A multi-level evaluation spanning controlled stress, Case Western Reserve University (CWRU) cross-load transfer, Paderborn current–vibration condition transfer, strict leave-one-physical-bearing-per-class-out testing, and a fault-class-held-out open-set replay audit.
- A repeated few-shot commissioning audit on 15 physical Paderborn bearings, quantifying performance as the target label budget increases from 12 to 96 records per fold, together with a controlled sensitivity analysis of the target-support sample weight.
- Deployment evidence that separates model-based sensing cost, local policy arithmetic, and a 32-run ESP32 telemetry pilot instead of conflating these three quantities.

## 2. Related Work

### 2.1. Edge Intelligence for Fault Monitoring

Vibration analysis remains a core technique for rotating machinery diagnosis because mechanical faults often produce characteristic spectral and impulsive patterns [14]. Deep learning has extended this tradition by learning discriminative features from raw signals or transformed representations [2,3]. Edge-oriented models further reduce latency and data transmission by performing diagnosis close to the device. For example, recent work has explored MobileNet-style edge diagnosis in wireless vibration sensor networks [4], while low-cost IoT predictive maintenance studies show the practical appeal of inexpensive vibration sensing [15].

### 2.2. Multi-Sensor Fusion and Feature Acquisition

Multi-sensor diagnosis can improve robustness by combining complementary signals. Recent works have used multimodal feature fusion or frequency-domain multi-sensor networks for bearing diagnosis [5,6]. Such models are valuable when all signals are available. However, they typically focus on how to combine measurements after acquisition. The acquisition decision itself is less emphasized: In many studies, vibration, acoustic, current, or other channels are assumed to be collected continuously. This assumption can be expensive in long-term low-cost monitoring.

### 2.3. Active Feature Acquisition and Dynamic Inference

Active feature acquisition optimizes which unobserved feature to query for each test instance. Prior approaches include jointly trained acquisition and classification policies, generative surrogates for estimating information gain, actor–critic sensing, and non-greedy acquisition oracles [7,8,9,16]. Recent stochastic-encoding methods reason over possible unobserved realizations without relying on a myopic one-step acquisition score [10]. These methods establish a general prediction–acquisition-cost framework, but they usually evaluate abstract feature groups rather than a physical screen–confirm sensor stack with fault-warning asymmetry.

Dynamic inference adapts neural computation after an input has already been observed. Early-exit and routing networks change depth or execution paths according to sample difficulty [11,12,13]. This line of work motivates cost–accuracy curves and per-sample routing, but reducing floating-point operations does not directly reduce the duty cycle of an unsampled vibration or audio channel. The present work therefore places the routing decision at the acquisition boundary and treats the full classifier as a replaceable downstream module.

### 2.4. Adaptive Sampling, Shift, and Commissioning

Adaptive sampling in sensor networks reduces energy consumption by changing acquisition according to the signal state or context [17]. A separate challenge appears after deployment: speed, mounting, sensor response, and physical device identity can shift the feature distribution. Source-free adaptation and uncertainty-guided open-set methods address this change from the representation-learning side [18,19]. Recent open-set bearing studies use contrastive domain adaptation or progressive domain separation to reject target-private classes [20,21]. Few-shot fault-diagnosis studies further show that limited target labels can be useful when zero-shot transfer is inadequate [22,23]. These representation-learning methods are complementary to acquisition control: Bayesian optimization can tune a policy offline, meta-learning can adapt the diagnostic model, and hybrid fusion combines channels after they have been sampled. Our controller instead decides whether the confirm channel must be acquired. It uses uncertainty and prototype distance only for escalation; the commissioning experiment separately measures how target labels repair the confirm stage. This separation avoids attributing classifier adaptation gains to the sensing policy.

## 3. Materials and Methods

### 3.1. Resource-Aware Acquisition Problem

For each monitoring window, let $x_{c}$ denote low-power features from current and temperature sensors, and let $x_{h}$ denote high-information features from vibration and acoustic sensors. The full feature vector is $x=[x_{c},x_{h}]$. The label *y* belongs to one of five states: healthy, bearing wear, rotor imbalance, electrical anomaly, and thermal blockage. The task is to predict a warning label while reducing the number of windows for which $x_{h}$ must be acquired.

Two classifiers are trained. The cheap classifier $f_{c}\left(x_{c}\right)$ runs on every window and outputs class probabilities $p_{c}$. The full classifier $f_{h}\left(x\right)$ runs only when the trigger policy activates vibration and acoustic sensing. Both classifiers are lightweight ridge-softmax classifiers in the experiment so that this paper can focus on the edge acquisition policy rather than a heavy backbone. In computational terms, the policy is a budgeted feature-acquisition layer placed before the full diagnostic model.

### 3.2. Risk, Uncertainty, and Prototype Memory

The cheap classifier provides an abnormality risk:

(1) $$r=1-p_{c}\left(y=\mathrm{healthy}|x_{c}\right).$$

It also provides a probability margin:

(2) $$m=p_{\left(1\right)}-p_{\left(2\right)},$$

where $p_{\left(1\right)}$ and $p_{\left(2\right)}$ are the largest and second-largest cheap-classifier probabilities. A small margin indicates uncertainty.

To detect cheap-feature drift, class prototypes are calculated in the standardized cheap feature space. For class *k*, the prototype is $\mu_{k}$, and the within-class spread is $s_{k}$. The normalized prototype distance is

(3) $$d=\min_{k}\frac{{∥z\left(x_{c}\right)-\mu_{k}∥}_{2}}{s_{k}+\epsilon },$$

where z(·) is standardization, and ϵ is a small constant.

### 3.3. Edge Acquisition Policy

Let $a_{c}=I\left[\arg\max_{k}p_{c}\left(k\right)\neq \mathrm{healthy}\right]$ denote a cheap-stage alarm. The high-cost sensing channels are activated when the cheap stage raises alarms, when the feature vector is outside the prototype support, or when a risky screen is hard to trust:

(4) $$g\left(x_{c}\right)=I\left[a_{c}=1\;\vee \;d>\tau_{d}\;\vee \;\left(r>\tau_{r}\;\wedge \;\left(m<\tau_{m}\;\vee \;r>\tau_{H}\right)\right)\right].$$

If $g(x_{c})=1$, the full classifier predicts with x; otherwise, cheap classifier prediction is used. The hard-confirmation branch prevents a low-cost feature shift from yielding a high-confidence but unverified fault warning. The rule therefore has four computational roles: alarm confirmation, risk-aware escalation, uncertainty-aware acquisition, and prototype-based drift screening. It remains simple enough to run as an edge-side control policy before high-bandwidth sensor streams are sampled or processed, although the hard-confirmation branch can increase the duty cycle when the cheap stream is weak.

Equation (4) cannot detect an unknown event that changes only an unobserved high-cost channel. For applications requiring a finite blind-interval guarantee, we augment the event trigger with a periodic watchdog:

(5) $$g_{t}^{+}=g\left(x_{c,t}\right)\vee I\left[t-t_{\mathrm{last}}\geq H\right],$$

where $t_{\mathrm{last}}$ is the most recent full acquisition, and *H* is the maximum allowed number of cheap-only windows. This mechanism does not identify a novel class; it guarantees that the confirm stream is observed at least once every *H* windows. We evaluate H=10 with two-second windows, giving a worst-case window-alignment delay of 20 s for a fault with no cheap-channel signature. Cheap-visible alarms or prototype drift escalate in the current window and therefore have at most a one-window (2 s) alignment delay before sensor wake-up and feature extraction.

### 3.4. Resource and Latency Model

The primary hardware-independent resource metric is the full-stage activation rate *a*. To interpret activations under different sensor stacks without presenting assumptions as measured power, we use the following normalized per-window cost:

(6) $$C\left(a;\kappa \right)=1+a\kappa ,\;\;\;\;\;S\left(a;\kappa \right)=\frac{(1-a)\kappa }{1+\kappa },$$

where the cheap-stage cost is normalized to one, $\kappa =C_{h}/C_{c}$ is the incremental high-to-cheap acquisition-cost ratio, and *S* is the savings relative to always-full acquisition. The submitted scenario corresponds to κ=6.13. This ratio was selected as an illustrative engineering scenario, not estimated from the ESP32 pilot and not interpreted as sensor-rail energy. We therefore report activation as the primary quantity, express cost as a percentage of always-full acquisition, and repeat the calculation for κ∈{1,2,4,6.13,8,10} rather than treating one assumed coefficient pair as universal.

Recent hardware-aware edge studies report power, throughput, and latency jointly across embedded platforms [24]. This standard motivates our explicit separation between the present activation-based comparison and the direct sensor-rail measurements still required for deployment.

Reaction time is decomposed as

(7) $$T_{\mathrm{react}}=T_{\mathrm{align}}+T_{\mathrm{wake}}+T_{\mathrm{acq}}+T_{\mathrm{feat}}+T_{\mathrm{policy}}.$$

Only $T_{\mathrm{policy}}$ is measured in the present processor benchmark. The window-alignment term is bounded by one window for cheap-visible events and by *H* windows for high-channel-only events under the watchdog. Sensor wake-up, acquisition, and feature extraction remain platform-specific unmeasured terms and are not hidden inside the desktop arithmetic timing.

### 3.5. Few-Shot Confirm-Stage Commissioning

Confirmation-complete routing cannot compensate for a confirm-stage classifier that fails after a physical-device shift. We therefore treat limited labelled commissioning as a separate deployment phase. For each unseen target bearing, *k* labelled records are sampled independently from every operating condition and class, with k∈{1,2,4,8}. These support records are excluded from evaluation, and all remaining records form the query set. Source records and target support records train a fixed Extra Trees confirm-stage model; the submitted setting assigns target support samples a weight of 20 so that a small commissioning set is not overwhelmed by the larger source cohort.

This protocol measures a label–performance curve rather than claiming zero-shot domain generalization. Each of five outer folds holds out one healthy, one inner-race-damaged, and one outer-race-damaged physical bearing. For robustness to support selection, each label budget is repeated with five independently sampled support sets within every target condition and class. The resulting 100 calibrated fits keep bearing identity separated between source and target and keep support records separated from query records. To test the balancing heuristic, the complete protocol is repeated for support weights {1,5,10,20,40,80}. Support draws and model random seeds are held identical across weights, so this ablation changes only target-sample weighting.

**Figure 1:**
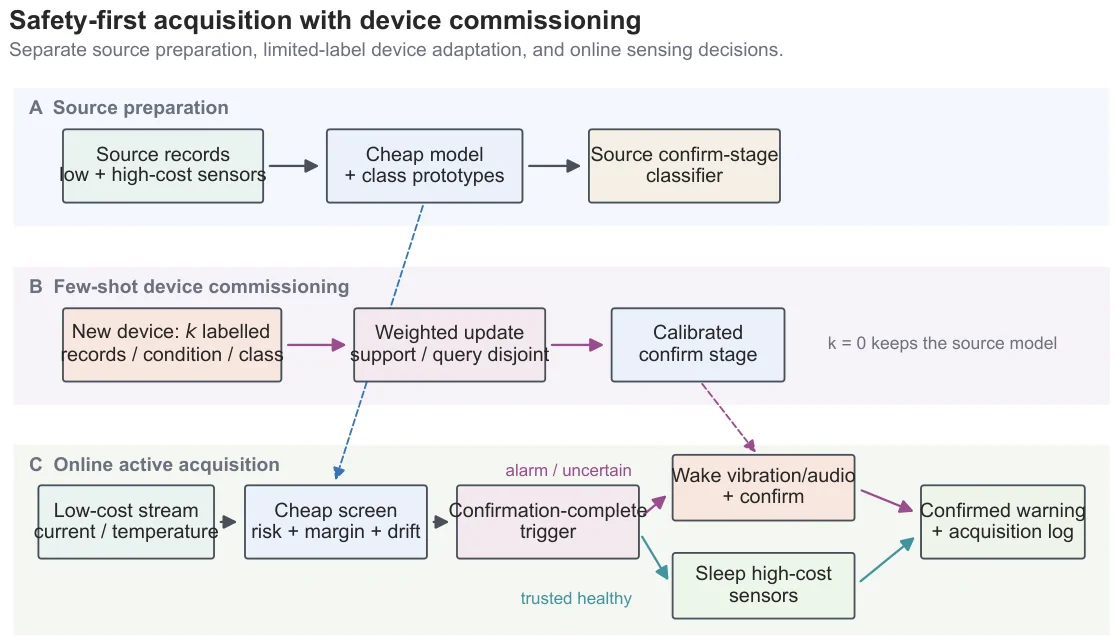
Safety-first acquisition and commissioning workflow. (**A**) Source records train the cheap screen, class prototypes, and confirm-stage classifier. (**B**) A limited labelled support set from a new device can update the confirm stage while remaining disjoint from query records; with k=0, the source model is retained. (**C**) During online monitoring, low-cost signals screen every window and high-cost vibration/audio channels are acquired only for alarms, uncertainty, or prototype drift. Blue dotted arrows transfer the source cheap model and prototypes to online screening; purple dotted arrows transfer the commissioned confirm stage and indicate escalation to confirmation; green/teal arrows denote the trusted-healthy sleep path.

Figure 1 summarizes source preparation, few-shot device commissioning, and online active acquisition.

## 4. Experimental Setup

### 4.1. Synthetic Benchmark

Because the purpose is to evaluate the sensing policy under controlled conditions, a reproducible synthetic benchmark was generated rather than being presented as a real campus deployment. The benchmark includes five classes: healthy, bearing wear, rotor imbalance, electrical anomaly, and thermal blockage. Each sample contains 15 features: six low-power current/temperature features and nine vibration/audio features.

The training set is class-balanced and generated under nominal conditions. The monitoring streams are generated with a healthier class prior to reflect continuous monitoring, with 520 healthy windows and 110 windows for each fault class per device. Five stress conditions are evaluated: nominal, speed shift, high noise, sensor drift, and combined stress. The fixed random seed is 20260609. The training set contains 4915 windows, the validation set contains 1385 windows, and each stress-condition test stream contains 3840 windows.

### 4.2. CWRU Cross-Load Evaluation Protocol

To reduce reliance on the synthetic benchmark, an additional public-data evaluation uses the Case Western Reserve University Bearing Data Center 12 kHz drive-end subset [25]. Four classes are retained: normal baseline, 0.007-inch inner-race fault, 0.007-inch ball fault, and 0.007-inch outer-race fault. The evaluation spans the four official motor-load settings (0, 1, 2, and 3 HP). For each of four folds, recordings from three loads supply chronological 70/30 training and validation partitions, while all windows from the remaining motor load are held out for testing. To keep class and load contributions balanced, 58 non-overlapping 2048-sample windows are retained from every recording.

This public dataset contains vibration rather than the current/temperature/acoustic configuration of the synthetic benchmark. It is therefore not a replacement for the multi-sensor setting. Instead, it tests the same screen-confirm logic under a public vibration protocol: the cheap stage uses low-rate time-domain features extracted from every 16th sample, while the full stage uses high-resolution time-domain and spectral features from the complete vibration window.

### 4.3. Paderborn Multimodal Condition-Transfer Protocol

An external multimodal stress test uses the Paderborn University Bearing Data Center [26]. Three bearing states are retained: healthy K001, inner-race damage KI04, and outer-race damage KA04. The dataset provides synchronized motor-current and vibration signals across four operating conditions. The cheap stage uses phase-current features, whereas the confirm stage uses phase-current plus vibration features. In each leave-one-condition-out fold, the first 14 recordings from each source condition are used for training, and the remaining six are used for validation; all 20 recordings from the held-out condition are tested. Because the same bearing identity appears across conditions, this experiment tests operating-condition transfers rather than unseen-bearing generalization. It is deliberately retained as a stress test: a policy that safely escalates when current screening becomes unreliable is useful, but it does not by itself solve a weak full-stage classifier under a large speed shift.

The strict identity audit expands this boundary to 15 physical bearings: K001–K005; KI01, KI03, KI05, KI07, and KI08; and KA01, KA03, KA05, KA06, and KA07. Five outer folds each hold out one physical bearing from every class across all four operating conditions, giving 240 target records per fold. No target record enters source-only fitting, normalization, checkpoint selection, or hyperparameter selection. The confirm-stage representation uses each four-second current–vibration record and its vibration envelope, interpolated to a measured-speed shaft-order grid from 0.25 to 80 orders. Fixed logistic-regression, RBF-SVM, and Extra Trees models are reported, together with a raw dual-branch gated CNN audit. Bearing identifiers, condition names, record indices, and target labels are not model inputs.

The open-set audit uses the same 15 physical bearings but removes one complete fault class from source training and threshold selection. Inner-race damage and outer-race damage are held out in turn. In each case, five outer folds additionally reserve one healthy bearing, one known-fault bearing, and one unseen-fault bearing for testing; source training uses records 1–14 from the remaining known-class bearings, and source-only validation uses records 15–20. The cheap model receives current features, the confirm model receives current plus vibration features, and neither model receives bearing identity, operating condition, record number, or target labels. Always-full sensing, an entropy gate, a source-trained learned error gate, the safety-first rule, and the safety-first rule with an H=10 watchdog are compared. The experimental unit for aggregate performance is the fault-type-by-physical-bearing fold (n=10), not an individual window.

Algorithmic reactions are evaluated by a controlled offline replay. For every held-out fault type, outer fold, and operating condition, all windows from the held healthy bearing are followed by all windows from the held unseen-fault bearing in official record order, giving 40 healthy-to-unseen step-transition streams. This construction isolates acquisition responses following an event that was absent from training; it is not a natural degradation chronology and does not estimate mechanical fault-propagation time. Delay is counted in acquisition-decision windows from the transition to the first confirm-stage activation. A 10,000-resample bootstrap over the 40 streams gives a descriptive 95% interval for mean delay.

The labelled commissioning protocol then samples *k* records per target condition and class from the same held-out bearings, where k∈{1,2,4,8}. This corresponds to 12, 24, 48, or 96 support labels per outer fold. All remaining target records are queries. Five independent support draws are evaluated for every fold and budget. The protocol therefore measures adaptation to an already installed device, not a diagnosis of a completely unseen bearing without calibration.

To identify whether the full-stage boundary arises partly from label-free covariate shifts rather than the acquisition rule, a commissioning ablation reuses the source-trained ridge weights but standardizes target features with a target-buffer mean and standard deviation. The target labels are never supplied to normalization or prediction. Buffers contain 24, 48, 96, or 192 full-feature windows (5%, 10%, 20%, or 40% of the 480-window target set). Rather than randomly resampling windows, the buffer follows the official recording order: The first recording from each of the three held target bearings, then the second recording from each, and so on. This creates a deterministic “fleet commissioning” benchmark under a new operating condition. It is not a natural chronological trace from one physical device, and it is not an energy-saving policy by itself.

### 4.4. Baselines and Metrics

Six policies are compared:

- **Always-Full**: vibration and acoustic sensing are active for every window.
- **Cheap-Only**: Only current and temperature features are used.
- **Fixed-Periodic**: Full sensing is activated once every three windows.
- **Random-Budget**: Full sensing is activated randomly with a budget comparable to a low-activation policy.
- **Static-Risk**: Full sensing is triggered only by abnormality risk.
- **Proposed**: Full sensing is triggered by a cheap-stage alarm, prototype distance, or the risk-margin rule.

The Paderborn trigger ablation additionally includes a learned error gate. A logistic model is trained only on source-validation records to predict whether the cheap classifier will be wrong; its decision threshold is selected on source validation and then frozen for the held-out condition. This provides a representative learning-based acquisition baseline without using target-condition labels. The reported metrics are accuracy, Macro-F1, false alarm rate, missed fault rate, full-sensor activation rate, normalized sensing cost, relative resource savings, and latency.

### 4.5. Joint Threshold, Unknown-Shift, and Cost Audits

Manual selection of three thresholds is inconvenient in deployment. We therefore evaluate a joint validation grid with $\tau_{r}\in \left\{0.2,\ldots ,0.8\right\}$, $\tau_{m}\in \left\{0.05,0.10,0.20,0.30,0.40\right\}$, and prototype-distance quantiles {0.70,0.80,0.90,0.96}. For an operator-selected resource penalty λ, the configuration maximizes

(8) $$J_{\lambda }=\mathrm{MacroF}1-\lambda a-0.10\;\mathrm{MR},$$

on source validation data, where *a* is the activation and MR is the missed-fault rate. The selected configuration is then evaluated once on the combined-stress test stream. This converts three manual thresholds into one interpretable safety-resource preference λ; a lower λ prioritizes conservative sensing, whereas a higher λ accepts more missed-fault risks to reduce activation.

Unknown behavior is audited at two levels. First, nominal healthy windows are perturbed in three reproducible ways: a shift visible in the cheap channels, a shift confined to high-cost channels, and a mixed shift. These perturbations isolate trigger mechanisms and are not claims of representative industrial fault physics. Second, the Paderborn fault-class-held-out protocol tests real public current–vibration records from a damage type absent from all training and tuning. At both levels, a non-healthy output from the closed-set confirm classifier is reported only as a generic known-class warning, not as correct unknown-class identification.

Resource conclusions are recomputed over κ∈{1,2,4,6.13,8,10} using Equation (6). This ratio analysis answers how the observed activation transfers to sensor stacks with different acquisition costs while keeping direct hardware power measurement outside the evidence boundary.

### 4.6. Local Compute-Overhead Benchmark

To check whether the adaptive trigger creates a meaningful computation burden, an execution-optimized Python 3.12.10 implementation of each policy was timed on the combined-stress stream. The same trained classifiers, prototypes, and trigger thresholds were used. Unlike the batch evaluation routine, the timing implementation runs the full classifier only on triggered windows for adaptive policies.

Each policy was warmed up for 20 passes and then timed for 160 repeated passes over 3840 windows. The benchmark measures only local CPU policy logic and lightweight classifier arithmetic. It excludes sensor wake-up, analog-to-digital conversion, feature extraction, radio transfer, operating-system scheduling, and embedded firmware overhead. Therefore, it is reported as a reproducible compute-overhead check rather than as hardware power validation.

### 4.7. ESP32 Hardware Telemetry Pilot

To move beyond a purely simulated sensing setup, a small real-device telemetry pilot was collected with an ESP32-WROOM-32 development board and a fan/blower device. The sensor stack included an ACS712-05B current sensor, a DS18B20 temperature sensor, a DA213B triaxial accelerometer, and an INMP441 microphone. The ACS712 was placed in the fan supply path; its measurement is therefore fan-load current, not ESP32 or sensor-rail current. The current, temperature, vibration, and audio sampling rates were 50 Hz, 1 Hz, 800 Hz and 8000 Hz, respectively.

The pilot contains four recorded condition codes, denoted C0–C3. Each code was repeated eight times. Each run lasted 60 s and was divided into non-overlapping 2 s windows, giving 30 windows per run and 960 windows in total. The spreadsheet supplied with this pilot records run-level summaries rather than raw per-window sensor streams. Therefore, this experiment is used as a hardware telemetry and feasibility check, not as a full hardware diagnostic-accuracy validation. C0 is the baseline, and C3 is explicitly marked as a 30% airflow-blocked condition. The physical interventions for C1 and C2 were not documented, so they are retained only as condition codes and are not assigned a mechanistic interpretation.

## 5. Results

### 5.1. Main Performance-Cost Comparison

Table 1 reports average results across all five stress conditions. Always-full sensing obtains the highest Macro-F1, but it acquires high-cost features for every window. Cheap-only sensing has the lowest modeled resource cost but loses detailed fault-warning performance. Fixed-periodic and random-budget policies reduce high-cost acquisition, but their decisions are not guided by diagnostic uncertainty.

**Figure 2:**
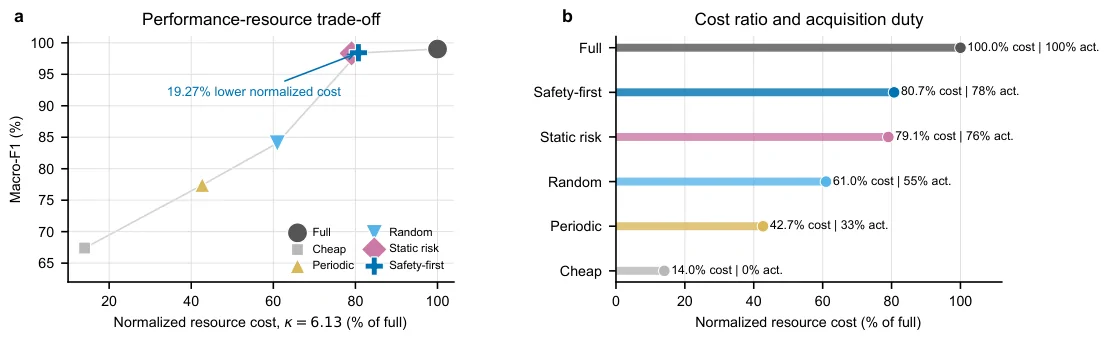
Policy comparison in terms of Macro-F1, activation, and normalized resource cost. Marker area in panel (**a**) scales with activation. Panel (**b**) ranks policies by normalized resource cost; endpoint labels report both cost and full-stage activation. Cost uses the stated κ=6.13 scenario and is expressed relative to always-full acquisition rather than as measured energy.

The proposed confirmation-complete policy achieves 98.42% Macro-F1 with a 77.59% full-feature activation rate. Under the illustrative κ=6.13 scenario, its normalized cost is 80.73% of always-full acquisition, a 19.27% reduction, while the Macro-F1 difference from always-full sensing is 0.60 percentage points. Relative to static-risk triggering, the proposed rule reduces missed faults from 1.68% to 1.48% but has a slightly higher duty cycle because every cheap-stage fault alarm is explicitly confirmed. This is a deliberate safety–resource trade-off rather than evidence that the proposed policy dominates every lower-duty trigger. Figure 2 visualizes this performance–resource trade-off and the component contributions to normalized cost.

### 5.2. Performance Under Simulated Stress Conditions

**Figure 3:**
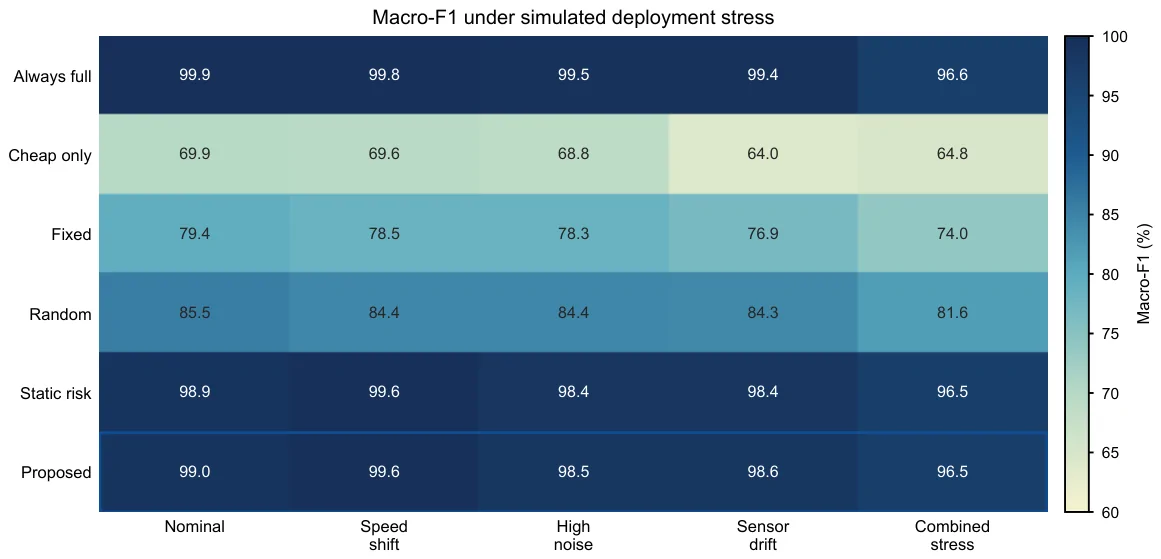
Macro-F1 under simulated deployment stress conditions using a fixed seed of 20260609. The proposed method tracks the always-full reference more closely than cheap-only and fixed-periodic baselines; this controlled benchmark is not a repeated real-device robustness estimate.

Table 2 and Figure 3 summarize the Macro-F1 value under each stress condition. The proposed method remains close to the always-full upper bound under nominal, speed-shift, high-noise, and sensor-drift conditions. Under combined stress, Macro-F1 decreases to 96.46%, consistent with the more difficult setting and with the elevated cheap-stage false-alarm tendency. Even in this condition, the proposed method remains substantially stronger than cheap-only and fixed-periodic policies.

### 5.3. Activation Behavior

**Figure 4:**
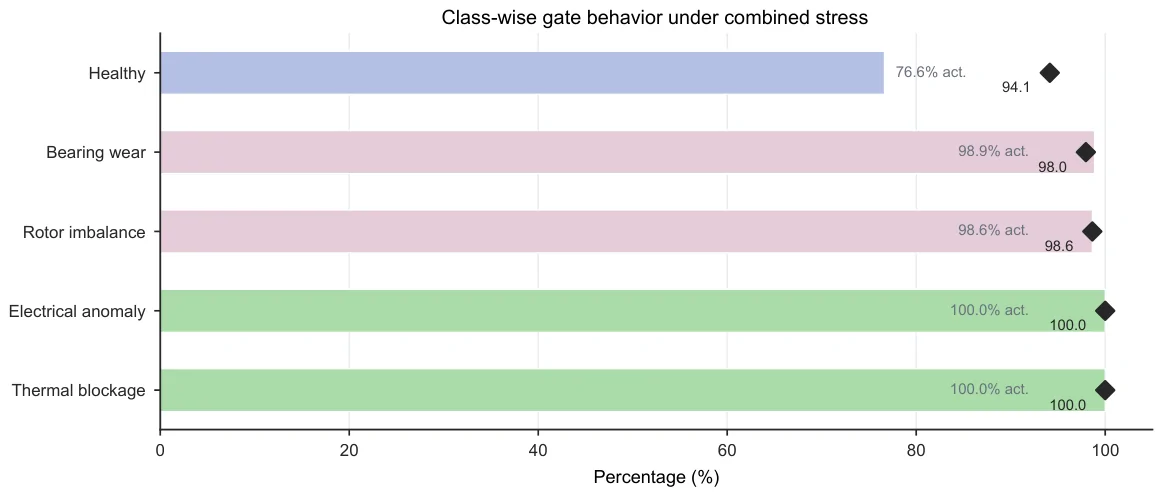
Full-sensor activation rate by true class under combined stress. The confirmation-complete policy routes nearly all fault alarms to the high-information stage and also confirms a subset of drifted healthy windows; black diamonds show class accuracy after confirmation.

Figure 4 shows activation under combined stress. The hard-confirmation branch activates the full stage for 98.86–100.00% of fault windows and for 76.64% of healthy windows. The healthy-window activation is the explicit cost of treating a low-cost alarm as a request for confirmation rather than as a final warning; it suppresses errors that a cheap stage would otherwise report with high confidence.

### 5.4. Joint Threshold, Unknown-Shift, and Resource Sensitivity

**Figure 5:**
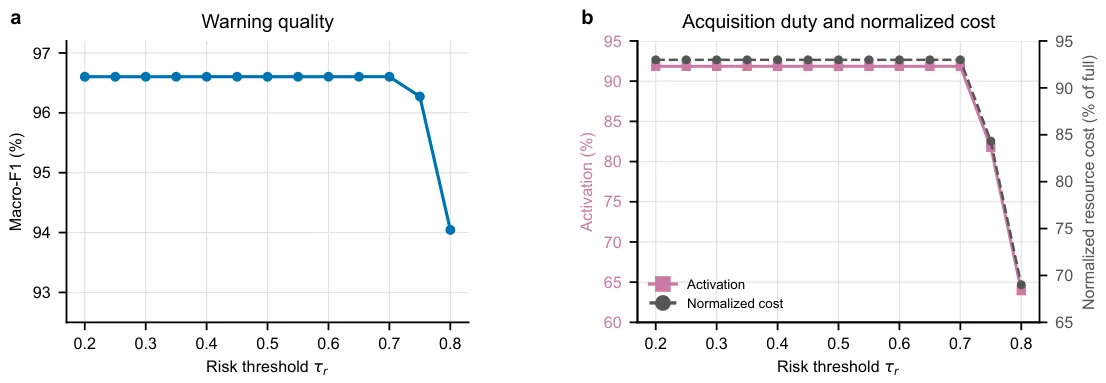
Sensitivity of the proposed sensing policy to the risk threshold. (**a**) Macro-F1 remains stable through $\tau_{r}=0.70$ and decreases at stricter settings. (**b**) Full-stage activation and normalized resource costs are shown on separate axes.

The risk threshold is not treated as a universal constant. A one-dimensional sweep from 0.20 to 0.80 shows the expected trade-off: lower thresholds increase high-cost activation and improve warning reliability, while higher thresholds reduce acquisition duty but increase the chance of missed or coarse predictions. Figure 5 reports the corresponding warning-quality and resource-cost sensitivity.

The joint grid provides a simpler operational rule: select a single resource penalty λ and obtain all three thresholds from source validation. Table 3 shows that λ=0.05–0.15 gives conservative configurations with 96.56–96.58% test Macro-F1, 97.73–99.22% activation, and no missed faults in the combined-stress stream. At λ=0.75, activation falls to 66.17%, but Macro-F1 falls to 94.21%, and missed faults rise to 6.93%. Thus, the tuning procedure exposes rather than hides the safety price of lower sensing duty.

Table 4 separates unknown-shift escalation from unknown-class recognition. Cheap-visible and mixed perturbations activate the confirm stage for 100.0% and 95.8% of windows, respectively. The high-channel-only perturbation leaves the cheap stream unchanged; the base policy therefore activates only 49.2% of windows and contains a 16-window inactive run. The stateful H=10 watchdog reduces the maximum inactive run to nine windows with a small activation increase to 49.5%. These outcomes show the fail-safe advantage precisely: the controller can bound access to the confirm channel, but a closed-set confirm classifier still cannot name an unseen class.

Finally, the resource-ratio sweep preserves the direction of the result without relying on one power assumption. At the observed activation a=0.7759, the relative savings is 11.21% for κ=1, 17.93% for κ=4, 19.27% for the submitted κ=6.13, and 20.37% for κ=10. Direct sensor-rail measurements would be required to choose the correct κ for a specific board.

### 5.5. Public CWRU Cross-Load Validation

**Figure 6:**
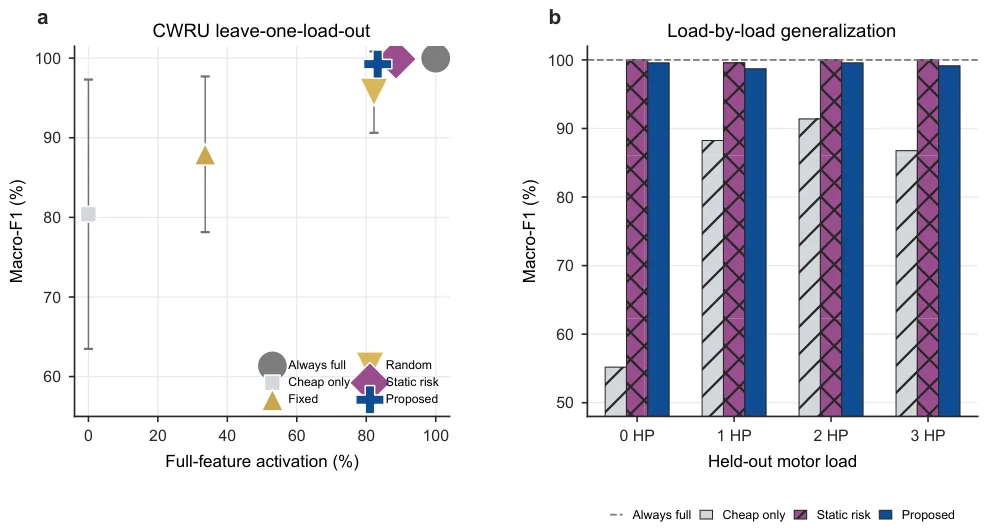
CWRU cross-load public-data validation. (**a**) Mean Macro-F1 versus full-feature activation across four leave-one-load-out folds; error bars show fold standard deviations. (**b**) Macro-F1 for each held-out motor load. The proposed policy remains near the always-full upper bound while reducing activation relative to static-risk triggering.

Table 5 and Figure 6 report the CWRU leave-one-load-out evaluation. Always-full feature extraction reaches 100.00% Macro-F1 in all four folds. Cheap-only features fall to 80.40 ± 16.91% Macro-F1, confirming that the low-rate screen is not reliable for cross-load diagnoses. The proposed policy achieves 99.25 ± 0.41% Macro-F1 with 83.30% full-feature activation and 14.32% scenario-modeled resource savings. It uses 5.28 percentage points less full-feature activation than static-risk triggering at a 0.65-point Macro-F1 difference. The per-load panel shows that the 0 HP holdout is the most difficult transfer condition, motivating the conservative confirmation branch.

### 5.6. Paderborn Multimodal Operating-Condition Stress Test

Table 6 reports the independent Paderborn current–vibration experiment and its trigger-component ablation. Current-only screening is weak under the leave-one-condition-out protocol (24.38 ± 6.54% Macro-F1). Reduced-duty trigger variants yield 23.35–39.46% scenario-modeled resource savings on average, but their Macro-F1 falls to 60.15–77.60% and their failure modes differ across operating conditions. The learned error gate also reaches only 71.24 ± 38.81% Macro-F1. Consequently, the confirmation-complete policy conservatively activates the vibration-confirm stage for all windows. It obtains the same 91.08 ± 17.55% Macro-F1 as always-full sensing rather than an apparent saving. The lowest source-only full-stage result occurs when the 900 rpm condition is held out (64.75% Macro-F1). Thus, this experiment supports the intended fail-safe behavior of the acquisition controller but simultaneously identifies rotating-speed domain generalization in the full classifier as a separate unsolved problem.

The separate physical-identity audit is substantially harder and is reported below. Its purpose is to distinguish a controller that escalates safely from a confirm-stage model that actually transfers to a new device.

**Figure 7:**
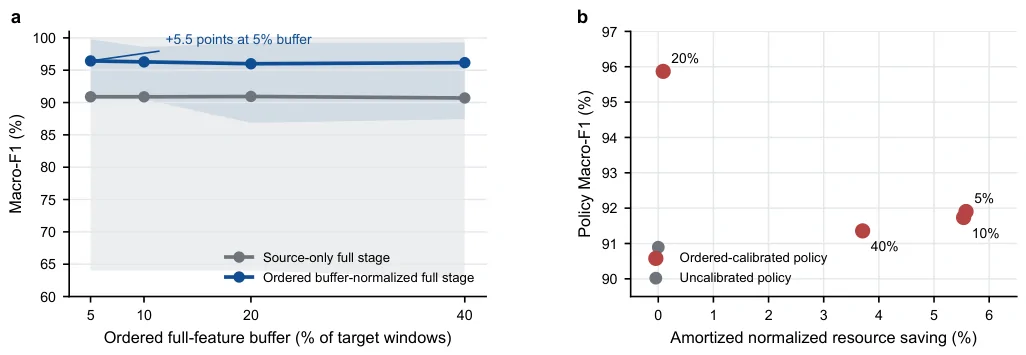
Ordered unlabeled commissioning calibration on the Paderborn condition-transfer test. (**a**) Mean full-stage Macro-F1 over four held-out operating conditions as an ordered target buffer grows; shaded bands show the range of condition means, not confidence intervals. (**b**) Macro-F1 versus amortized normalized resource savings for the calibrated confirmation policy; labels give buffer fraction. The buffer follows official recording numbers across the three target bearing files and contains full-stage features. It therefore represents deterministic fleet commissioning, not a natural single-device chronological stream.

Figure 7 shows that a deterministic ordered buffer can recover much of this shift without inspecting target labels. With the first 24 full-feature windows (5%), the buffer-normalized full stage reaches 96.44 ± 4.26% Macro-F1 compared with 90.90 ± 17.92% for the source-only model on the same remaining windows. The recovery is again concentrated in the 900 rpm holdout. When the normalized cheap and full stages are used by the proposed policy, the 5% buffer yields 91.91 ± 7.74% Macro-F1 with a 93.15% post-commissioning activation rate and 5.58% normalized resource savings after charging every buffer window as a full acquisition. Larger buffers do not monotonically improve the policy trade-off because the policy remains conservative. This is evidence of a potentially useful commissioning operation, not a solution to streaming domain generalization or a universal target-normalization gain.

### 5.7. Strict Unseen-Bearing and Label-Budget Audit

**Figure 8:**
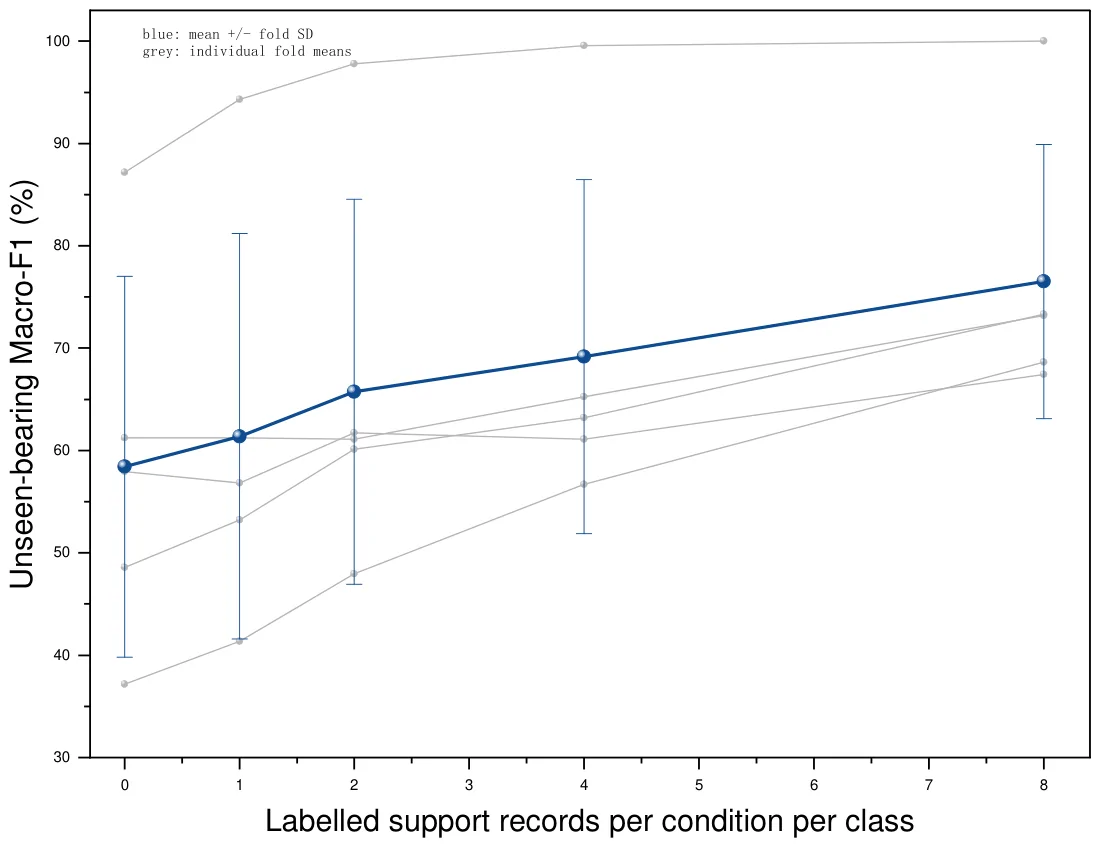
Label–performance curve under strict physical-bearing separation. The blue curve shows the mean Macro-F1 across five outer folds; error bars show the standard deviation of fold means. Grey curves show individual fold means. For each non-zero budget, five support sets are sampled independently within every target condition and class, and support records are excluded from the query set.

Table 7 and Figure 8 report the expanded 15-bearing identity audit. Under source-only transfers, the strongest fixed order-spectrum model is Extra Trees at 58.42 ± 18.60% Macro-F1. Logistic regression and RBF-SVM reach 56.72 ± 16.27% and 51.78 ± 12.96%, respectively. A raw dual-branch current–vibration CNN reaches 46.46 ± 23.97% at the record level, indicating that additional model capacity alone does not remove the identity shift under this protocol.

Limited labelled commissioning produces a monotonic aggregate improvement. Averaged over five outer folds and five random support draws per fold, 12, 24, 48, and 96 support labels yield 61.39%, 65.74%, 69.17%, and 76.52% Macro-F1. At the largest tested budget, every outer-fold mean exceeds its source-only Extra Trees result, and the overall gain is 18.10 percentage points. The grey fold trajectories in Figure 8 also reveal substantial device heterogeneity: one fold approaches saturation with very few labels, whereas the hardest bearing set remains much lower. The commissioning curve therefore supports a bounded operational conclusion—small target support sets can repair part of the device shift, but neither zero-shot transfer nor uniform target performance is established.

### 5.8. Target-Support Weight Sensitivity

**Figure 9:**
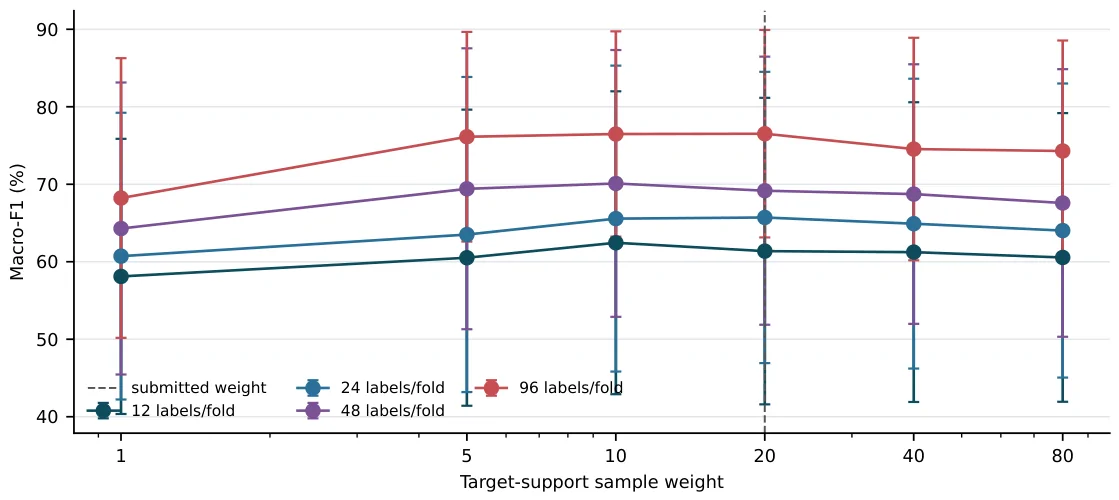
Sensitivity of strict Paderborn few-shot commissioning to target-support sample weight. Curves show means across five outer folds and five support draws per fold; error bars show the standard deviation of outer-fold means. The vertical dashed line marks the submitted weight of 20. Support samples and model seeds are identical across weights.

Figure 9 tests whether the submitted target-support weight of 20 determines the commissioning conclusion. With 12 labels per fold, the mean Macro-F1 increases from 58.11% at weight 1 to 62.45% at weight 10 and 61.38% at weight 20. With 96 labels, the corresponding values are 68.22%, 76.49%, and 76.52%. Weights 10 and 20 differ by at most 1.08 percentage points across the four label budgets, whereas weight 80 reduces the mean Macro-F1 to 60.56–74.30%. The result supports a moderate 10–20 weighting range rather than a uniquely optimal fixed value. The unchanged support draws across weights ensure that these differences are not caused by resampling.

### 5.9. Fault-Class-Held-Out Open-Set Replay

**Figure 10:**
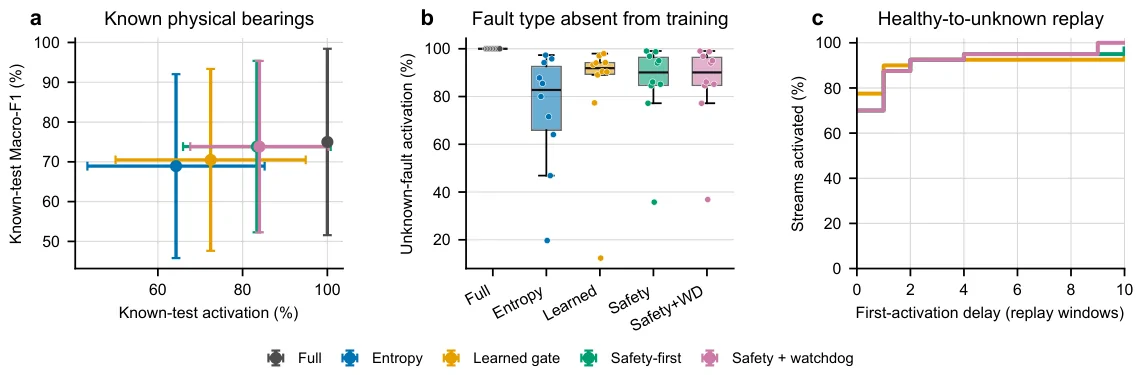
Fault-class-held-out public-data audit. (**a**) Known-class Macro-F1 versus activation; points show fold means, and error bars show standard deviations across ten fault-type-by-physical-bearing folds. (**b**) Confirm-stage activation on the held damage type; boxes show interquartile ranges, center lines show medians, whiskers extend to 1.5 interquartile ranges, and points are the ten folds. (**c**) Empirical cumulative fraction of 40 held-bearing-by-condition healthy-to-unseen replay streams reaching their first confirm-stage activation. The replay follows official record order after a constructed step transition and is not a natural degradation trace.

Table 8 and Figure 10 report the public-data audit in which an entire damage type is absent from training and tuning. Across the ten fault-type-by-bearing folds, the always-full closed-set classifier reaches 74.99±23.42% Macro-F1 on held physical bearings from the two known classes. The safety-first policy reaches 73.83±21.54% at 83.38±17.43% activation. Thus, the routing rule remains close to the available full-stage reference on known classes, although the large between-bearing spread confirms that physical-identity transfer remains difficult.

On the held damage type, the entropy gate, learned error gate, and safety-first rule activate for 74.28±24.89%, 83.61±25.69%, and 85.25±18.84% of windows, respectively. These are escalation rates, not unknown-class accuracy. The closed-set full classifier emits a non-healthy known-class warning for 71.64±23.05% of unseen-fault windows under always-full sensing and 60.86±20.20% under safety-first routing; neither number establishes correct fault-type identification.

The healthy-to-unseen replay gives the acquisition response independently of a class label. The safety-first rule first activates after a mean of 0.925 replay windows (bootstrap 95% interval 0.275–1.775), with 95% of 40 streams activated within five windows and 97.5% within ten. Adding the H=10 watchdog gives a mean delay of 0.825 windows (0.275–1.525) and activates all 40 streams within ten windows. The largest inactive run observed across the ten unseen-fault folds falls from 13 windows without the watchdog to nine windows with it, matching the design bound. Because the replay concatenates records from different physical bearings, it demonstrates algorithmic escalation after a controlled step transition rather than natural degradation timing.

### 5.10. Local Compute-Overhead Check

**Figure 11:**
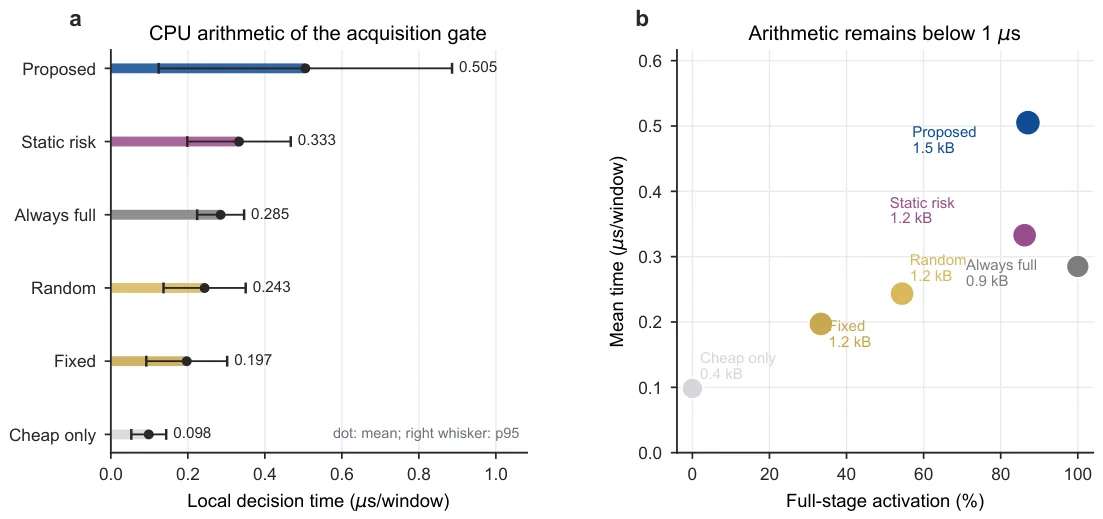
Local CPU arithmetic benchmark. In panel (**a**), dots show mean time, and right whiskers show p95 time over 160 repeated passes; panel (**b**) relates mean arithmetic time to full-stage activation and model-state size. Sensor wake-up, acquisition, feature extraction, and embedded power are excluded.

Table 9 and Figure 11 report the local CPU timing benchmark. Because the full-stage classifier is deliberately small, always-full inference is already inexpensive in pure arithmetic. The proposed policy is therefore not faster than always-full sensing in this narrow timing test; it performs cheap screening, prototype-distance calculation, and conditional full-stage inference. However, its absolute decision time remains sub-microsecond on the test machine, with a mean of 0.505 μs and a p95 of 0.886 μs per window. This supports the intended interpretation: the main cost saving comes from reduced sensor acquisition rather than classifier arithmetic speed.

Table 10 places this arithmetic result in the full reaction-time decomposition. With two-second non-overlapping windows, a cheap-visible event has a mean alignment delay of 1 s and a worst-case delay of 2 s. A high-channel-only event under the H=10 watchdog has a mean alignment delay of 10 s and a worst-case delay of 20 s. The measured 0.505 μs policy arithmetic is negligible relative to these window bounds, but the unmeasured sensor wake-up, acquisition, and feature-extraction terms prevent a claim of end-to-end embedded latency.

### 5.11. Hardware Telemetry Pilot

**Figure 12:**
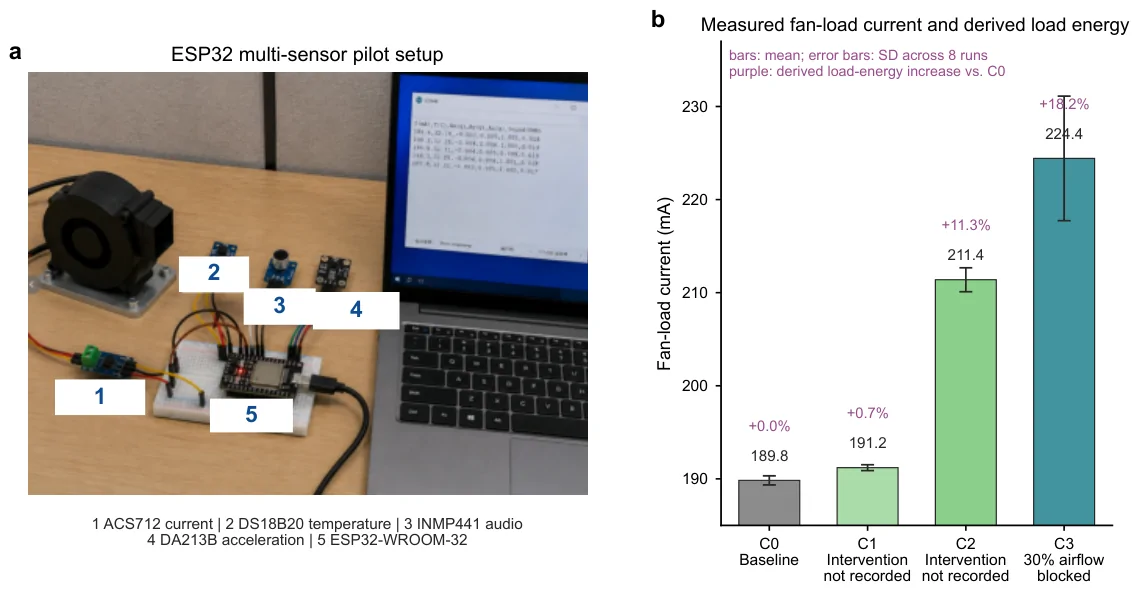
ESP32-based hardware telemetry pilot. (**a**) Fan/blower setup with ACS712-05B fan-current, DS18B20 temperature, DA213B acceleration, and INMP441 audio sensing. (**b**) Bars show mean run-level fan current, and error bars show sample standard deviation across eight runs; purple labels show derived fan-load energy increase for a 12 V supply and 2 s windows. These values are not ESP32 or sensor-rail power. C1 and C2 are retained as condition codes because their physical interventions were not recorded.

Table 11 and Figure 12 summarize the ESP32 fan telemetry pilot. The measured fan-load current and derived per-window load energy remain stable in the baseline condition. Relative to C0, code C2 increases the mean derived load energy by 11.35%, and the documented 30% airflow-blocked C3 condition increases it by 18.22%. Because the physical interventions for C1 and C2 were not recorded, these codes are reported descriptively rather than interpreted as specific load or fault mechanisms. These measurements do not prove hardware diagnostic accuracy or sensing-energy savings; they establish acquisition-stack feasibility and run-level fan-current differences.

## 6. Discussion

The results support a modular view of edge diagnosis in which acquisition control and confirm-stage generalization are distinct problems. The confirmation-complete rule makes an explicit safety choice: A low-cost alarm is not emitted as a final warning until a high-information stage has checked it. This rule kept the synthetic Macro-F1 within 0.60 percentage points of always-full sensing while avoiding full acquisition on 22.41% of windows. Under Paderborn condition transfers, the same conservatism activated every window when current screening was unreliable. The controller is therefore a routing layer, and its warning quality remains upper-bounded by the confirm-stage classifier.

The learned error gate provides the closest learning-based comparator in the public multimodal test. It predicts cheap-stage errors from source validation data, but its 71.24% Macro-F1 and 24.92% missed-fault rate show that a learned trigger can fail after condition shift. The proposed rule offers a different theoretical property rather than a universal accuracy advantage. Every cheap-stage non-healthy decision is confirmed by construction, and the watchdog guarantees a full acquisition at least once every *H* windows. A probabilistic gate has no corresponding guarantee unless these constraints are added explicitly. Bayesian optimization could tune the validation objective in Equation (8), meta-learning could adapt the confirm classifier, and hybrid fusion could combine channels after acquisition. These approaches are therefore complementary to, rather than substitutes for, the proposed acquisition constraint.

The joint threshold audit converts three manually selected thresholds into one resource penalty. Low λ values preserved a zero missed-fault rate at the cost of near-continuous acquisition, whereas λ=0.75 reduced activation to 66.17% and increased missed faults to 6.93%. The two unknown-event audits expose the corresponding safety boundary. In the mechanism test, cheap-visible and mixed perturbations were escalated in nearly every window, but a high-channel-only shift could remain invisible to the event trigger. In the stricter public-data test, the safety-first rule activated for 85.25% of windows from a physical bearing and damage type absent from training. Its mean replay delay was below one acquisition-decision window, but one base-policy stream required more than ten windows; the watchdog brought all 40 streams within the stated ten-window horizon and limited the observed inactive run to nine. This is a deterministic access guarantee, not open-set semantic recognition.

The two commissioning studies expose different forms of target access. Ordered unlabeled normalization partially repaired an operating-condition shift, whereas labelled support records improved strict cross-bearing transfer from 58.42% to 76.52% Macro-F1 at the largest tested budget. The support-weight audit shows that this trend is not tied to the submitted weight of 20: Weights 10 and 20 remain within 1.08 percentage points across all label budgets. Performance falls with no target emphasis and with excessive weight 80. The large between-fold spread nevertheless shows that commissioning burden is device-dependent rather than universal.

Several limitations define the evidence boundary. The main policy benchmark is synthetic, and the normalized resource coefficients are engineering scenario values. CWRU is vibration-only and validates screen–confirm logic rather than a complete multimodal stack. Paderborn covers one test rig; even the strict 15-bearing and fault-class-held-out audits contain only healthy and artificially damaged bearings. The constructed replay is a controlled step transition, not a run-to-failure chronology. The ESP32 pilot stores run-level summaries instead of synchronized raw streams. It therefore supports telemetry feasibility and fan-current differences, but not end-to-end hardware diagnostic accuracy or sensor-rail power. The cost-ratio sweep makes the resource conclusion less dependent on one coefficient pair, but it does not replace direct measurement. A complete deployment study still requires synchronized raw hardware signals, on-board trigger execution, direct low/high-channel power measurements, long-duration natural degradation streams, and unseen physical units.

## 7. Reproducibility Materials

The manuscript package retains the synthetic generator, CWRU and Paderborn data scripts, Paderborn trigger and calibration ablations, strict unseen-bearing and fault-class-held-out replay audits, expanded record-level order-spectrum and labelled-commissioning calibration audits, local compute benchmarks, hardware-pilot workbooks, setup images, source tables, plotting scripts, and collection templates. The synthetic benchmark uses a fixed seed of 20260609, and generated CSV files provide source data for all figures and tables. The open-set script uses a fixed seed of 20260729 and exports fold-level thresholds, predictions, activation statistics, and stream-level delays. CWRU files are downloaded from official Bearing Data Center URLs listed in the validation scripts. Paderborn data are obtained from the official KAt DataCenter under its CC BY-NC 4.0 license. The hardware-pilot summary is derived from the supplied spreadsheet and setup image. For journal submission, these files can be supplied in the Supplementary Materials to separate reproducible simulation, public-data evidence, hardware telemetry, and future raw-signal validation.

## 8. Conclusions

This paper presented resource-aware, safety-first active sensor acquisition for edge fault warning. Confirmation-complete routing reduced unnecessary high-cost acquisition when the confirm stage transferred and escalated conservatively when the cheap stream became unreliable. The controlled benchmark retained 98.42% Macro-F1 with 77.59% activation, while CWRU cross-load evaluation reached 99.25 ± 0.41% Macro-F1 at 83.30% activation. Strict Paderborn identity separation exposed the complementary adaptation problem: Source-only order-spectrum diagnosis reached 58.42 ± 18.60% Macro-F1, and repeated labelled commissioning increased it to 76.52 ± 13.39% with 96 support labels per fold. When a complete damage type and physical bearing were absent from training, the controller activated the confirm channel for 85.25±18.84% of unseen-fault windows; the watchdog brought every constructed replay stream to full acquisition within ten decision windows. Joint threshold selection, support-weight sensitivity, cost-ratio analysis, and the watchdog make the controller’s resource and safety assumptions explicit. The present evidence does not establish sensor-rail energy savings, raw-stream hardware diagnosis, unknown-class recognition, or universal cross-device transfer. It quantifies when reduced sensing is credible and when conservative acquisition or labelled commissioning is required.

## Figures and Tables

**Table 1:** Average performance and normalized resource cost across five simulated stress conditions. Cost is reported as a percentage of always-full acquisition for κ=6.13 and is not a power measurement.

Policy	Acc. (%)	F1 (%)	FA (%)	Miss (%)	Act. (%)	Norm. Cost (% Full)
Always-full	99.06	99.02	1.51	0.07	100.00	100.00
Cheap-only	71.87	67.40	24.41	13.27	0.00	14.03
Fixed-periodic	80.77	77.42	16.87	8.94	33.33	42.68
Random-budget	86.61	84.07	12.02	5.76	54.59	60.96
Static-risk	98.45	98.34	1.28	1.68	75.65	79.07
Proposed	98.52	98.42	1.32	1.48	77.59	80.73

**Table 2:** Macro-F1 (%) under simulated stress conditions.

Policy	Nom.	Speed	Noise	Drift	Combined
Always-full	99.90	99.79	99.47	99.38	96.56
Cheap-only	69.87	69.58	68.79	63.95	64.79
Fixed-periodic	79.44	78.47	78.29	76.91	73.99
Static-risk	98.86	99.57	98.39	98.35	96.53
Proposed	98.97	99.60	98.50	98.59	96.46

**Table 3:** Joint threshold selection by a single validation resource penalty.

λ	$\tau_{r}$	$\tau_{m}$	$q_{d}$	F1 (%)	Act. (%)	Miss (%)
0.05	0.70	0.10	0.70	96.56	99.22	0.00
0.15	0.70	0.10	0.96	96.58	97.73	0.00
0.35	0.60	0.05	0.96	96.28	87.42	0.85
0.55	0.60	0.05	0.96	96.28	87.42	0.85
0.75	0.80	0.05	0.96	94.21	66.17	6.93

**Table 4:** Mechanism audit under reproducible synthetic unknown shifts. “WD” denotes the H=10 watchdog, and rates are not unknown-class accuracy.

Perturbation	Alarm (%)	OOD (%)	Base Act. (%)	WD Act. (%)	Max Gap
Cheap-visible	100.0	100.0	100.0	100.0	0
High-channel-only	25.0	5.3	49.2	49.5	9
Mixed	39.0	61.3	95.8	95.8	2

**Table 5:** CWRU four-fold leave-one-motor-load-out validation. Values are mean ± standard deviation over held-out loads.

Policy	F1 (%)	Act. (%)	Savings (%)
Always-full	100.00±0.00	100.00	0.00
Cheap-only	80.40±16.91	0.00	85.71
Fixed-periodic	87.92±9.78	33.62	56.90
Random-budget	95.74±5.11	82.22	15.24
Static-risk	99.89±0.22	88.58	9.79
Proposed	99.25±0.41	83.30	14.32

**Table 6:** Paderborn synchronous current–vibration condition-transfer stress test and trigger ablation. Values are mean ± standard deviation across four held-out operating conditions.

Policy	F1 (%)	Act. (%)	Savings (%)	Miss (%)
Always-full	91.08±17.55	100.00	0.00	10.70
Cheap-only	24.38±6.54	0.00	85.71	45.86
Alarm-confirm	65.41±34.19	53.96	39.46	45.86
Margin-only	77.60±40.66	72.76	23.35	24.69
Prototype-only	60.15±16.49	56.72	37.10	21.41
Learned-error gate	71.24±38.81	63.44	31.34	24.92
Proposed	91.08±17.55	100.00	0.00	10.70

**Table 7:** Strict five-fold leave-one-physical-bearing-per-class-out Paderborn audit. Commissioning results first average five random support draws within each fold; reported deviations are then calculated across the five fold means.

Confirm-Stage Protocol	Labels/Fold	Macro-F1 (%)
Order-spectrum logistic regression	0	56.72±16.27
Order-spectrum RBF-SVM	0	51.78±12.96
Order-spectrum Extra Trees	0	58.42±18.60
Raw gated CNN (record level)	0	46.46±23.97
Weighted commissioning, k=1	12	61.39±19.81
Weighted commissioning, k=2	24	65.74±18.79
Weighted commissioning, k=4	48	69.17±17.30
Weighted commissioning, k=8	96	76.52±13.39

**Table 8:** Paderborn fault-class-held-out audit. Values are mean ± standard deviation across two held fault types and five physical-bearing folds (n=10). Unknown activation is an acquisition response, not unknown-class accuracy.

Policy	Known F1 (%)	Known Act. (%)	Unknown Act. (%)
Always-full	74.99±23.42	100.00±0.00	100.00±0.00
Entropy gate	68.92±23.11	64.29±20.92	74.28±24.89
Learned-error gate	70.49±22.86	72.46±22.44	83.61±25.69
Safety-first	73.83±21.54	83.38±17.43	85.25±18.84
Safety-first + H=10	73.82±21.54	83.96±16.32	85.36±18.52

**Table 9:** Local CPU policy-and-classifier timing on the combined-stress stream.

Policy	Act. (%)	Mean Time (μs/Window)	P95 Time (μs/Window)	State (kB)
Always-full	100.00	0.285	0.346	0.9
Cheap-only	0.00	0.098	0.144	0.4
Fixed-periodic	33.33	0.197	0.302	1.2
Random-budget	54.40	0.243	0.350	1.2
Static-risk	86.20	0.333	0.467	1.2
Proposed	87.06	0.505	0.886	1.5

**Table 10:** Reaction-time bounds for the proposed event trigger and optional watchdog.

Event Visibility	Mean Align. (s)	Worst Align. (s)	Policy Arithmetic (μs/Window)	Unmeasured Terms
Cheap-visible	1.0	2.0	0.505	wake, acquisition, features
High-channel-only	10.0	20.0	0.505	wake, acquisition, features

**Table 11:** ESP32 fan telemetry pilot. Load energy is calculated from measured fan-supply current, 12 V supply voltage, and a 2 s window.

Condition	Runs	Windows	Current (mA)	Energy (J/Window)
C0 baseline	8	240	189.84±0.49	4.556±0.012
C1, intervention unrecorded	8	240	191.20±0.32	4.589±0.008
C2, intervention unrecorded	8	240	211.39±1.29	5.073±0.031
C3, 30% airflow blocked	8	240	224.43±6.69	5.386±0.161

## Data Availability

The CWRU Bearing Data Center data are publicly available online: https://engineering.case.edu/bearingdatacenter (accessed on 9 June 2026). The Paderborn Bearing Data Center data are publicly available online under the repository’s stated terms: https://mb.uni-paderborn.de/en/kat/research/bearing-datacenter (accessed on 10 July 2026). The processed source tables, analysis scripts, fixed-seed synthetic and open-set run reports, fold-level thresholds, replay delays, local timing results, and hardware-pilot workbook and setup image underlying this article are included in the Supplementary Materials supplied with the submission. Raw synchronized hardware sensor streams were not collected; the hardware pilot contains run-level summaries, as stated in this manuscript.

## Supplementary Materials

The following supporting information can be downloaded at: https://www.mdpi.com/article/10.3390/s26165065/s1, a reproducibility package containing processed source tables, experiment and figure-generation codes, machine-readable run reports, fold-level open-set and replay results, the hardware-pilot workbook and setup image, and editable figure source files. Raw CWRU and Paderborn records are not redistributed and can be obtained from their official repositories.
