# Supplementary material for: Resource-Aware Safety-First Active Sensor Acquisition with Few-Shot Commissioning for Edge Fault Warning
Source: Sensors (Basel). 2026 Aug 10;26(16):5065. doi: 10.3390/s26165065 (PMC13517865; doi:10.3390/s26165065)
Supplement: Supplementary file 1 [file sensors-26-05065-s001.zip › figures/calibration_buffer.pdf]

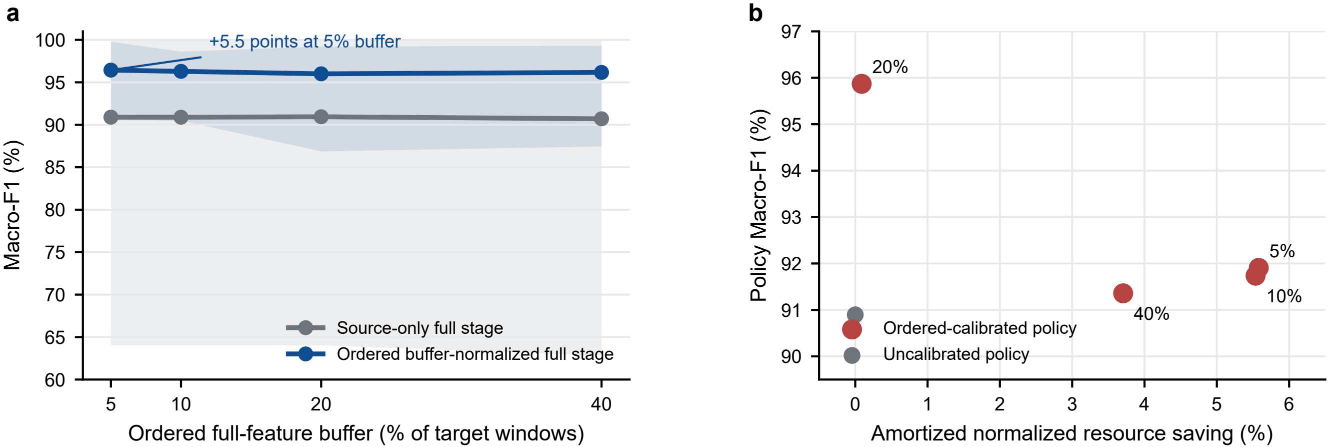

a
+5.5 points at 5% buffer
Macro-F1 (%)
100
95
90
85
80
75
70
65
60
5
10
20
40
Ordered full-feature buffer (% of target windows)
Source-only full stage
Ordered buffer-normalized full stage
b
Policy Macro-F1 (%)
97
96
95
94
93
92
91
90
0
1
2
3
4
5
6
Amortized normalized resource saving (%)
20%
5%
10%
40%
Ordered-calibrated policy
Uncalibrated policy
