# Supplementary material for: Resource-Aware Safety-First Active Sensor Acquisition with Few-Shot Commissioning for Edge Fault Warning
Source: Sensors (Basel). 2026 Aug 10;26(16):5065. doi: 10.3390/s26165065 (PMC13517865; doi:10.3390/s26165065)
Supplement: Supplementary file 1 [file sensors-26-05065-s001.zip › origin_figures/fig2_policy_comparison_origin.pdf]

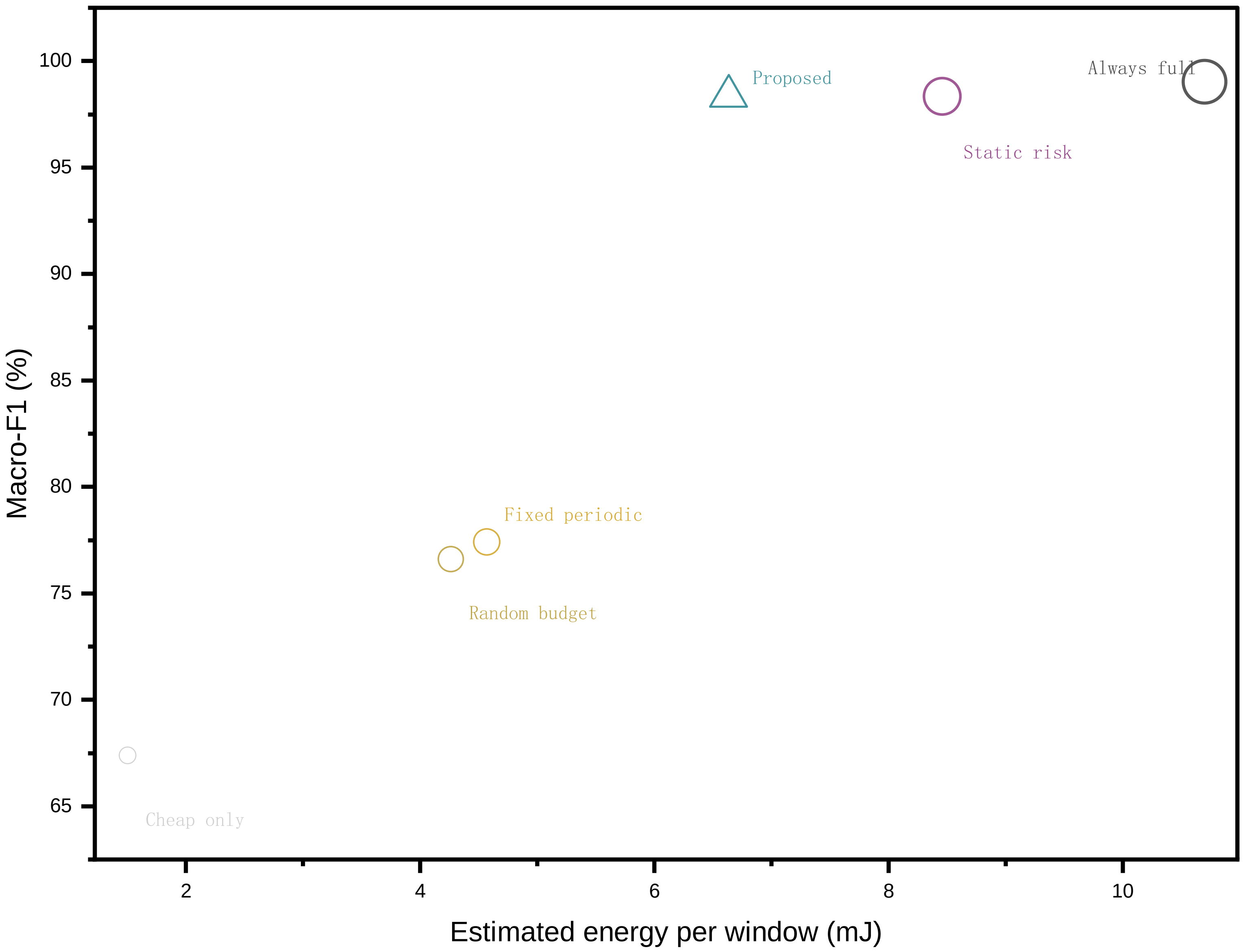

Proposed
Always full
Static risk
Fixed periodic
Random budget
Cheap only
100
95
90
85
80
75
70
65
2
4
6
8
10
Macro-F1 (%)
Estimated energy per window (mJ)
