# Supplementary material for: Resource-Aware Safety-First Active Sensor Acquisition with Few-Shot Commissioning for Edge Fault Warning
Source: Sensors (Basel). 2026 Aug 10;26(16):5065. doi: 10.3390/s26165065 (PMC13517865; doi:10.3390/s26165065)
Supplement: Supplementary file 1 [file sensors-26-05065-s001.zip › origin_figures/fig6_cwru_public_validation_origin.pdf]

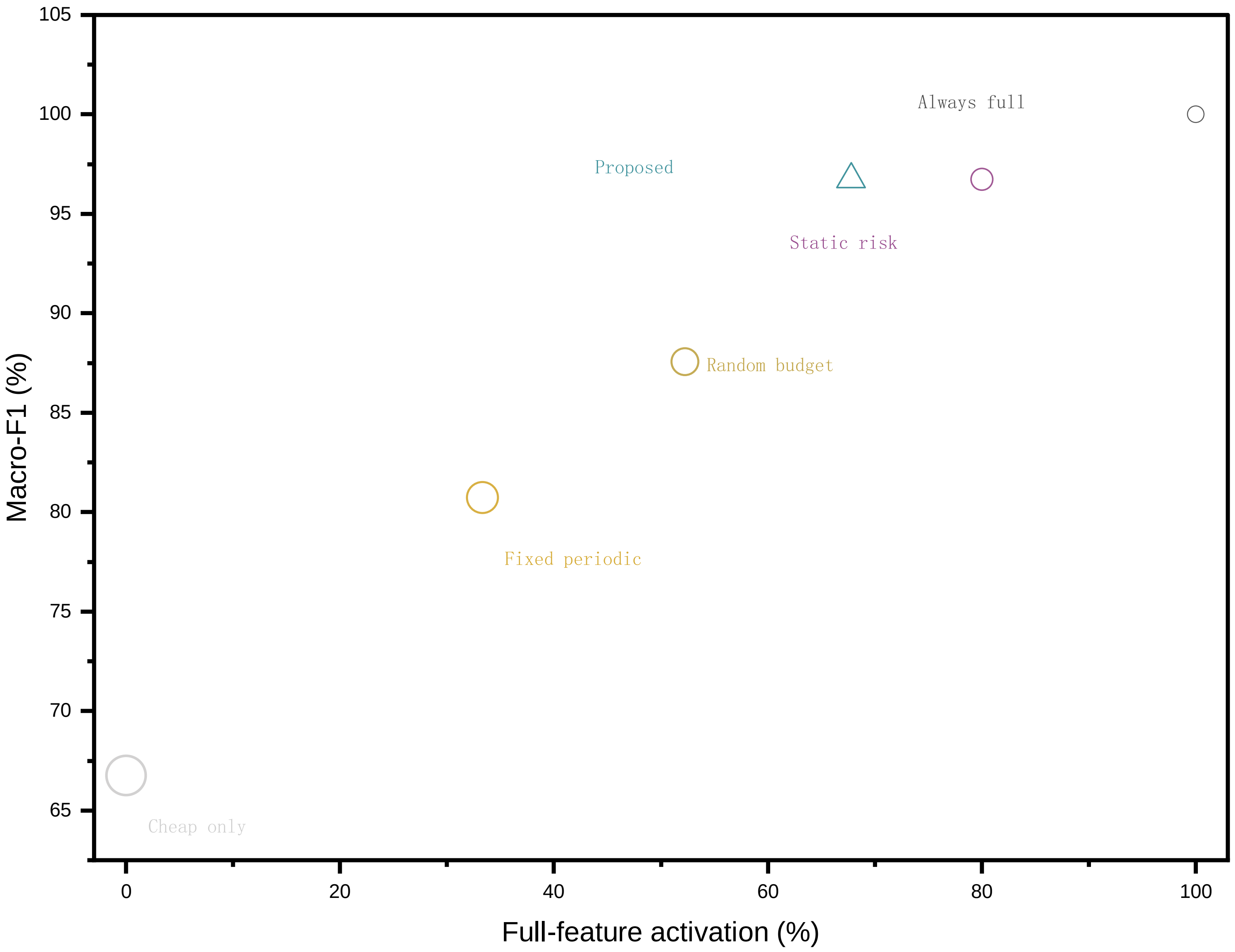

Always full
Proposed
Static risk
Random budget
Fixed periodic
Cheap only
Macro-F1 (%)
105
100
95
90
85
80
75
70
65
0
20
40
60
80
100
Full-feature activation (%)
