# Supplementary material for: Resource-Aware Safety-First Active Sensor Acquisition with Few-Shot Commissioning for Edge Fault Warning
Source: Sensors (Basel). 2026 Aug 10;26(16):5065. doi: 10.3390/s26165065 (PMC13517865; doi:10.3390/s26165065)
Supplement: Supplementary file 1 [file sensors-26-05065-s001.zip › origin_figures/fig7_edge_overhead_origin.pdf]

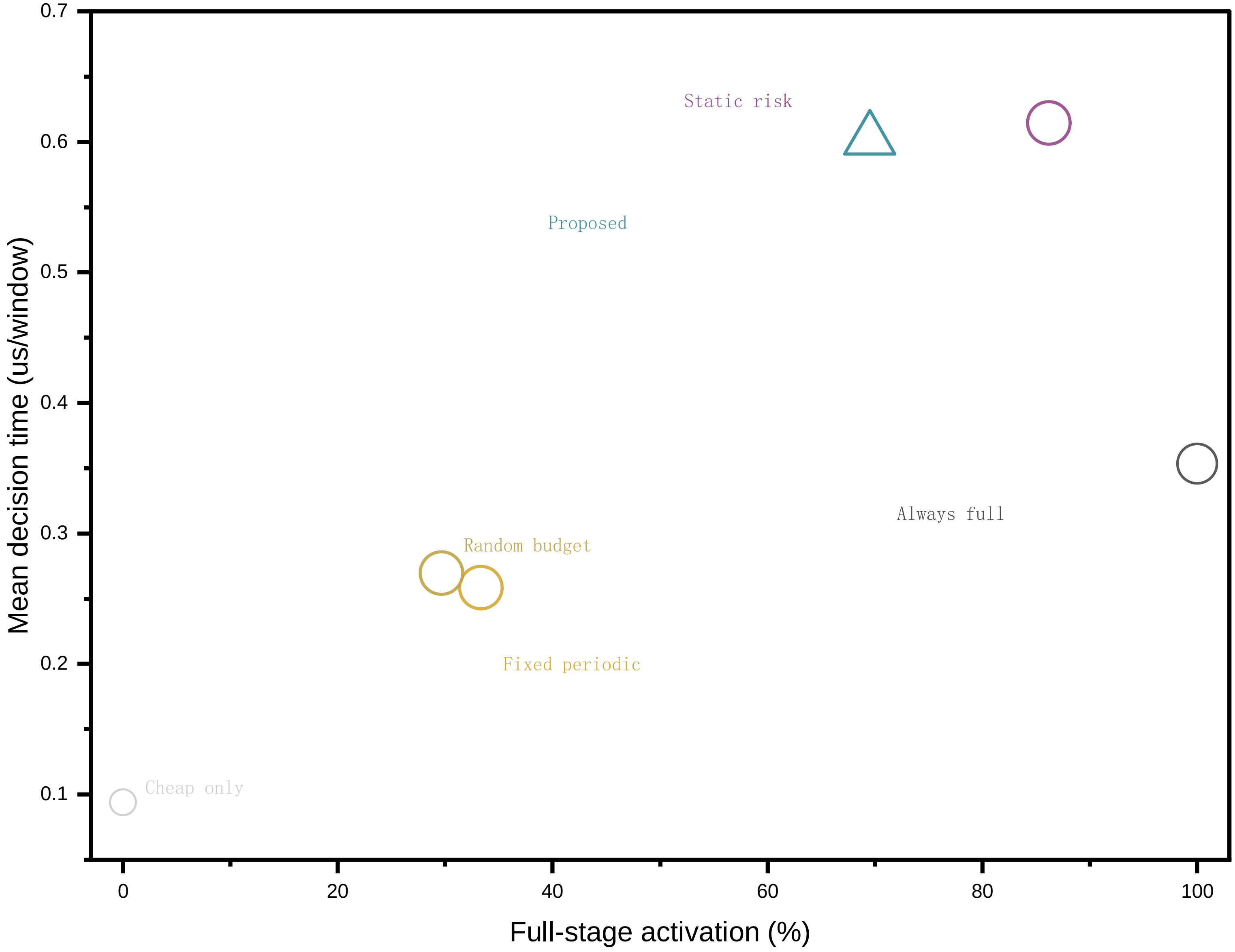

Static risk
Proposed
Always full
Random budget
Fixed periodic
Cheap only
Mean decision time (us/window)
Full-stage activation (%)
0.1
0.2
0.3
0.4
0.5
0.6
0.7
0
20
40
60
80
100
