# Supplementary material for: Resource-Aware Safety-First Active Sensor Acquisition with Few-Shot Commissioning for Edge Fault Warning
Source: Sensors (Basel). 2026 Aug 10;26(16):5065. doi: 10.3390/s26165065 (PMC13517865; doi:10.3390/s26165065)
Supplement: Supplementary file 1 [file sensors-26-05065-s001.zip › origin_figures/fig8_hardware_current_origin.pdf]

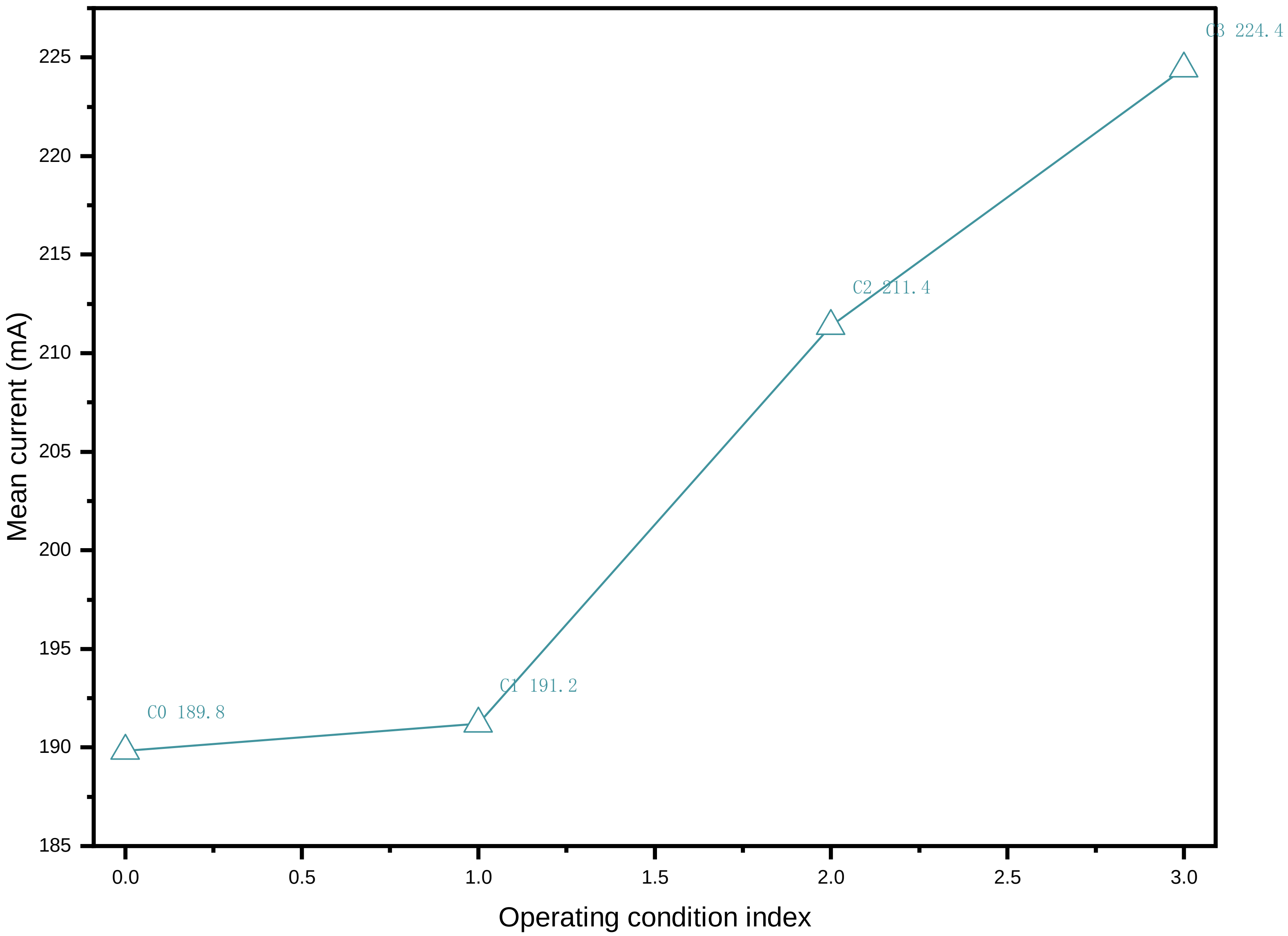

C0 189.8
C1 191.2
C2 211.4
C3 224.4
Mean current (mA)
185
190
195
200
205
210
215
220
225
0.0
0.5
1.0
1.5
2.0
2.5
3.0
Operating condition index
