# Supplementary material for: Resource-Aware Safety-First Active Sensor Acquisition with Few-Shot Commissioning for Edge Fault Warning
Source: Sensors (Basel). 2026 Aug 10;26(16):5065. doi: 10.3390/s26165065 (PMC13517865; doi:10.3390/s26165065)
Supplement: Supplementary file 1 [file sensors-26-05065-s001.zip › origin_figures/fig8_hardware_energy_origin.pdf]

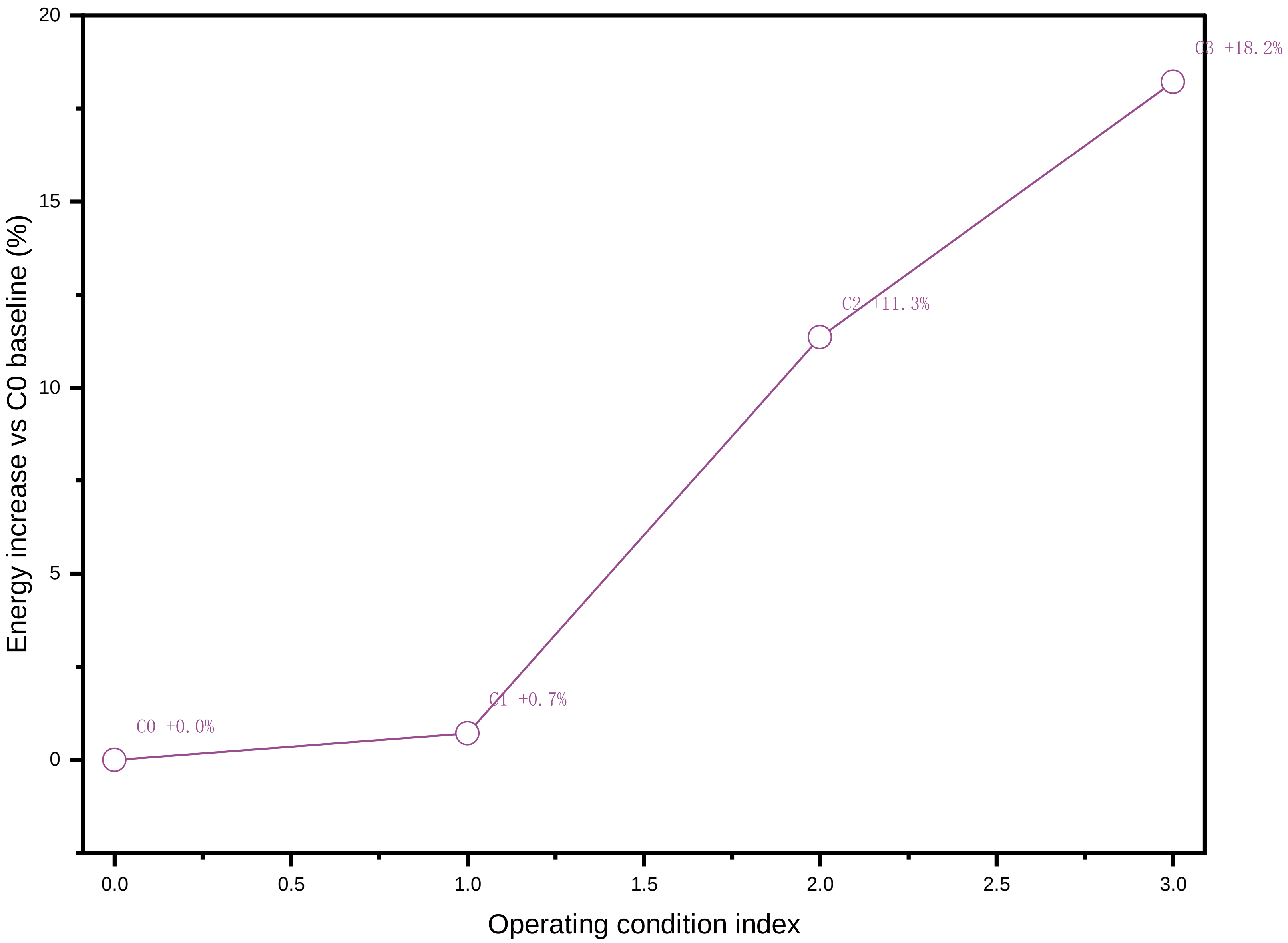

Energy increase vs C0 baseline (%)
20
15
10
5
0
0.0
0.5
1.0
1.5
2.0
2.5
3.0
Operating condition index
C0 +0.0%
C1 +0.7%
C2 +11.3%
C3 +18.2%
