# Supplementary material for: Resource-Aware Safety-First Active Sensor Acquisition with Few-Shot Commissioning for Edge Fault Warning
Source: Sensors (Basel). 2026 Aug 10;26(16):5065. doi: 10.3390/s26165065 (PMC13517865; doi:10.3390/s26165065)
Supplement: Supplementary file 1 [file sensors-26-05065-s001.zip › paper_rewriting_output/offline_v2/fig2_policy_comparison_v2.pdf]

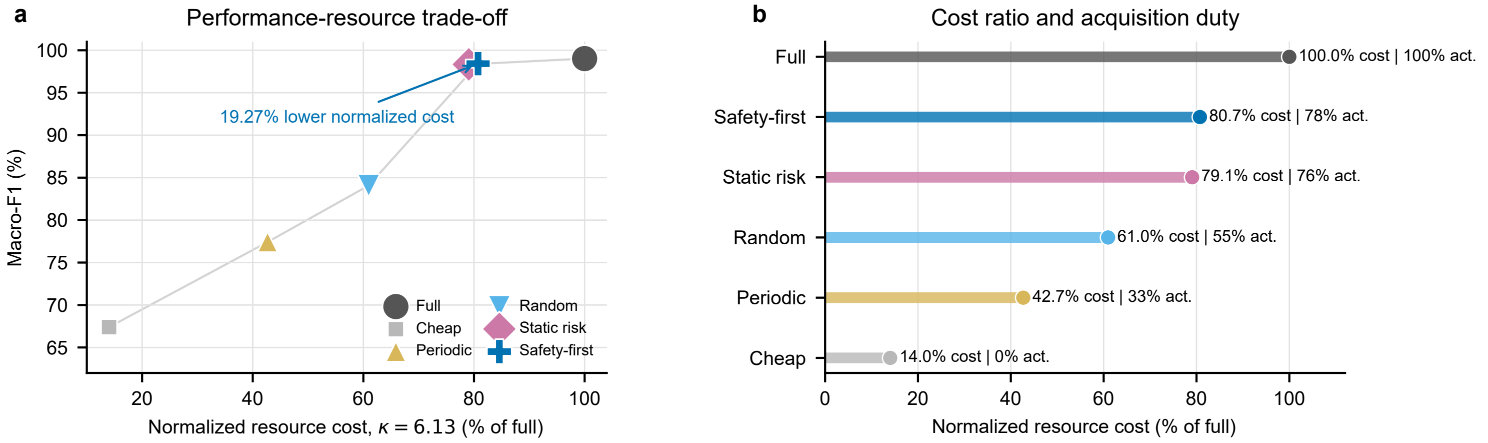
a
Performance-resource trade-off
19.27% lower normalized cost
Macro-F1 (%)
Normalized resource cost, κ = 6.13 (% of full)
Full
Cheap
Periodic
Random
Static risk
Safety-first
b
Cost ratio and acquisition duty
Full
Safety-first
Static risk
Random
Periodic
Cheap
100.0% cost | 100% act.
80.7% cost | 78% act.
79.1% cost | 76% act.
61.0% cost | 55% act.
42.7% cost | 33% act.
14.0% cost | 0% act.
Normalized resource cost (% of full)
