# Supplementary material for: Resource-Aware Safety-First Active Sensor Acquisition with Few-Shot Commissioning for Edge Fault Warning
Source: Sensors (Basel). 2026 Aug 10;26(16):5065. doi: 10.3390/s26165065 (PMC13517865; doi:10.3390/s26165065)
Supplement: Supplementary file 1 [file sensors-26-05065-s001.zip › paper_rewriting_output/offline_v2/fig5_threshold_sensitivity_v2.pdf]

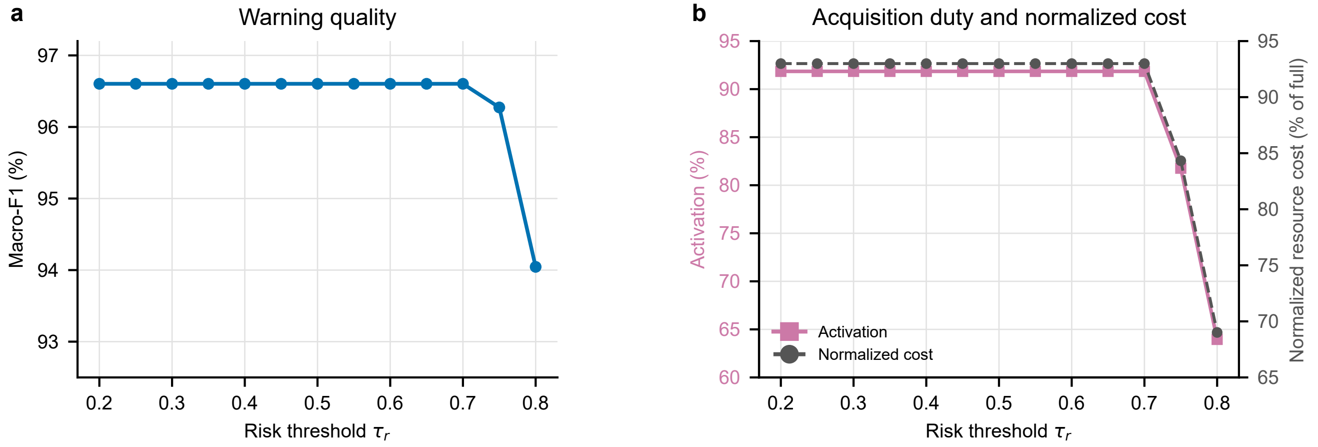

a
Warning quality
Macro-F1 (%)
97
96
95
94
93
0.2
0.3
0.4
0.5
0.6
0.7
0.8
Risk threshold τ_r
b
Acquisition duty and normalized cost
Activation (%)
95
90
85
80
75
70
65
60
Normalized resource cost (% of full)
Activation
Normalized cost
0.2
0.3
0.4
0.5
0.6
0.7
0.8
Risk threshold τ_r
