# Supplementary material for: Resource-Aware Safety-First Active Sensor Acquisition with Few-Shot Commissioning for Edge Fault Warning
Source: Sensors (Basel). 2026 Aug 10;26(16):5065. doi: 10.3390/s26165065 (PMC13517865; doi:10.3390/s26165065)
Supplement: Supplementary file 1 [file sensors-26-05065-s001.zip › paper_rewriting_output/offline_v2/fig9_paderborn_open_set_replay.pdf]

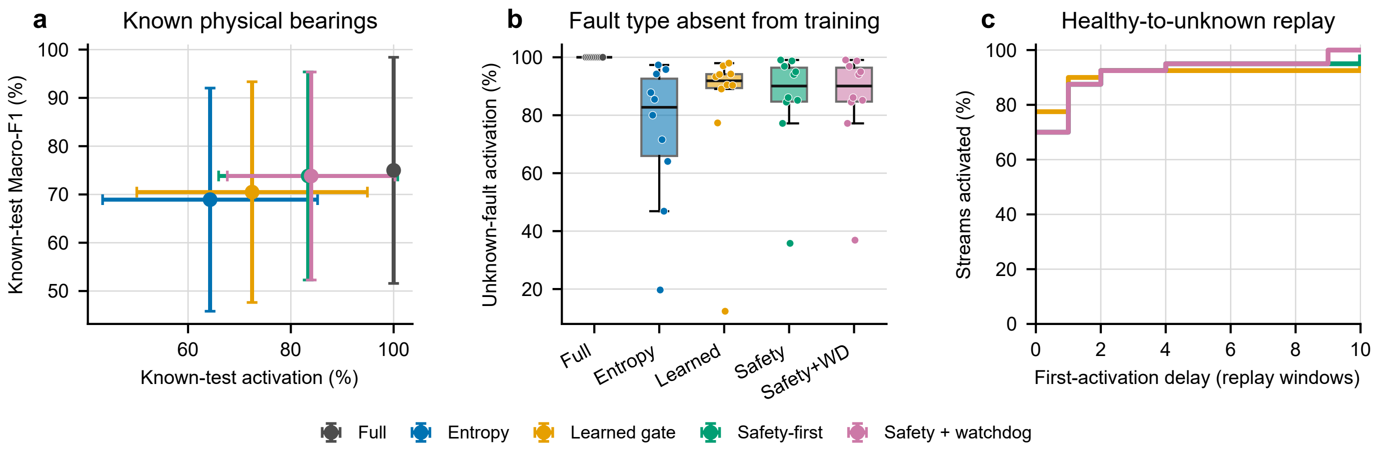

a
Known physical bearings
Known-test Macro-F1 (%)
100
90
80
70
60
50
60
80
100
Known-test activation (%)
b
Fault type absent from training
Unknown-fault activation (%)
100
80
60
40
20
Full
Entropy
Learned
Safety
Safety+WD
c
Healthy-to-unknown replay
Streams activated (%)
100
80
60
40
20
0
0
2
4
6
8
10
First-activation delay (replay windows)
Full
Entropy
Learned gate
Safety-first
Safety + watchdog
