# Supplementary material for: Resource-Aware Safety-First Active Sensor Acquisition with Few-Shot Commissioning for Edge Fault Warning
Source: Sensors (Basel). 2026 Aug 10;26(16):5065. doi: 10.3390/s26165065 (PMC13517865; doi:10.3390/s26165065)
Supplement: Supplementary file 1 [file sensors-26-05065-s001.zip › paper_rewriting_output/revision_round1/joint_threshold_sensitivity.pdf]

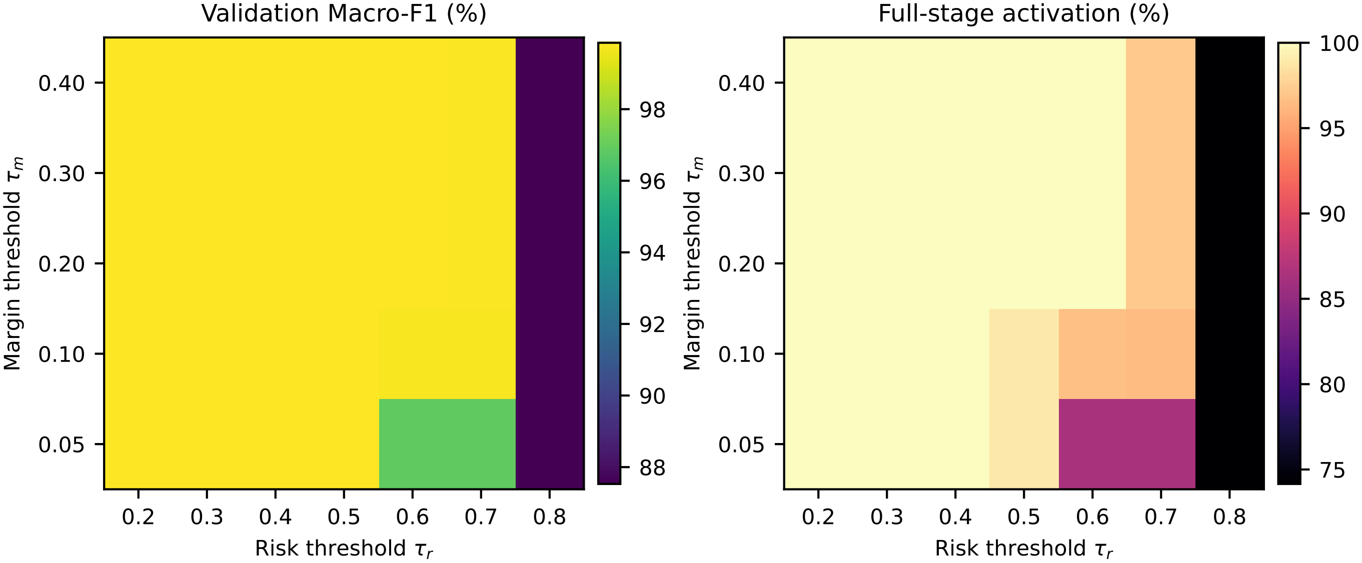

Validation Macro-F1 (%)
Full-stage activation (%)
Margin threshold τ_m
Risk threshold τ_r
0.40
0.30
0.20
0.10
0.05
0.2
0.3
0.4
0.5
0.6
0.7
0.8
98
96
94
92
90
88
100
95
90
85
80
75
