# Supplementary material for: Resource-Aware Safety-First Active Sensor Acquisition with Few-Shot Commissioning for Edge Fault Warning
Source: Sensors (Basel). 2026 Aug 10;26(16):5065. doi: 10.3390/s26165065 (PMC13517865; doi:10.3390/s26165065)
Supplement: Supplementary file 1 [file sensors-26-05065-s001.zip › paper_rewriting_output/revision_round1/support_weight_sensitivity.pdf]

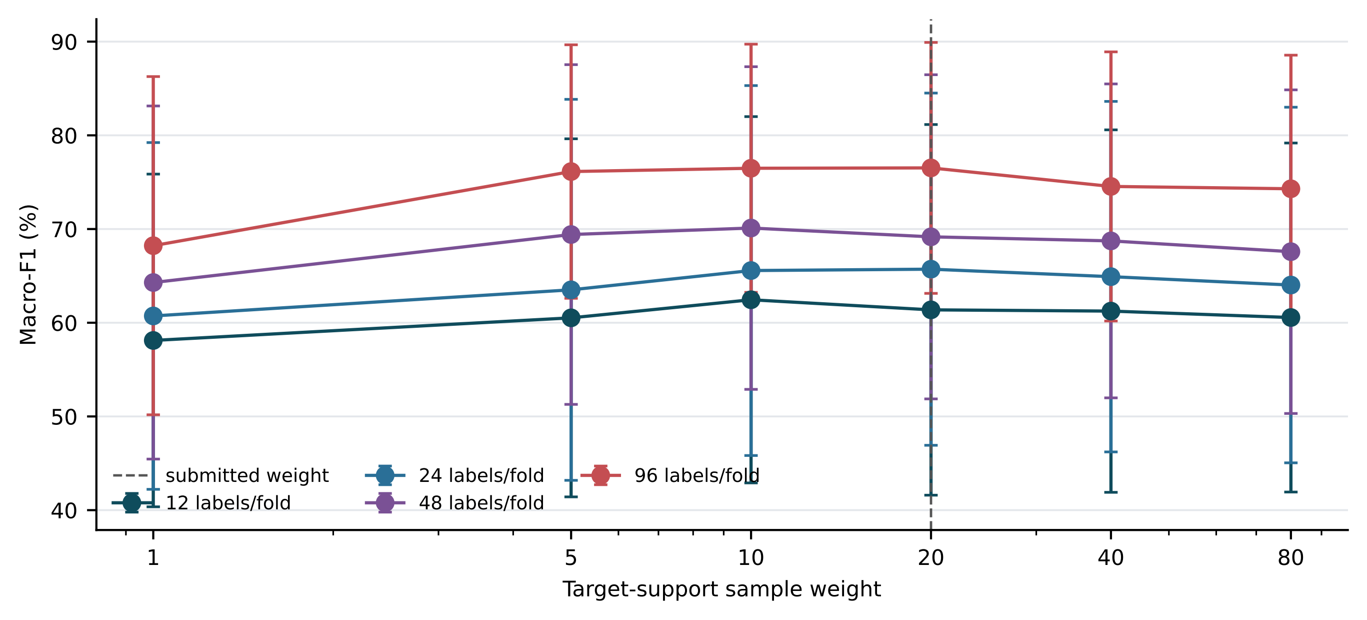

Macro-F1 (%)
90
80
70
60
50
40
1
5
10
20
40
80
Target-support sample weight
submitted weight
12 labels/fold
24 labels/fold
48 labels/fold
96 labels/fold
